# Expression of *Ciona intestinalis* AOX causes male reproductive defects in *Drosophila melanogaster*

**DOI:** 10.1186/s12861-017-0151-3

**Published:** 2017-07-03

**Authors:** Sina Saari, Ana Andjelković, Geovana S. Garcia, Howard T. Jacobs, Marcos T. Oliveira

**Affiliations:** 1 Institute of Biosciences and Medical Technology, Tampere University Hospital, University of Tampere, FI-33014 Tampere, Finland; 2 Departamento de Tecnologia, Faculdade de Ciências Agrárias e Veterinárias, Universidade Estadual Paulista “Júlio de Mesquita Filho”, Jaboticabal, SP 14884-900 Brazil; 3 Institute of Biotechnology, University of Helsinki, FI-00014 Helsinki, Finland

**Keywords:** Mitochondria, Respiratory chain, Spermatogenesis, Sperm competition

## Abstract

**Background:**

Mitochondrial alternative respiratory-chain enzymes are phylogenetically widespread, and buffer stresses affecting oxidative phosphorylation in species that possess them. However, they have been lost in the evolutionary lineages leading to vertebrates and arthropods, raising the question as to what survival or reproductive disadvantages they confer. Recent interest in using them in therapy lends a biomedical dimension to this question.

**Methods:**

Here, we examined the impact of the expression of *Ciona intestinalis* alternative oxidase, AOX, on the reproductive success of *Drosophila melanogaster* males. Sperm-competition assays were performed between flies carrying three copies of a ubiquitously expressed AOX construct, driven by the α-tubulin promoter, and wild-type males of the same genetic background.

**Results:**

In sperm-competition assays, AOX conferred a substantial disadvantage, associated with decreased production of mature sperm. Sperm differentiation appeared to proceed until the last stages, but was spatially deranged, with spermatozoids retained in the testis instead of being released to the seminal vesicle. High AOX expression was detected in the outermost cell-layer of the testis sheath, which we hypothesize may disrupt a signal required for sperm maturation.

**Conclusions:**

AOX expression in *Drosophila* thus has effects that are deleterious to male reproductive function. Our results imply that AOX therapy must be developed with caution.

**Electronic supplementary material:**

The online version of this article (doi:10.1186/s12861-017-0151-3) contains supplementary material, which is available to authorized users.

## Background

In the animal kingdom, the alternative mitochondrial respiratory chain is widely represented, as illustrated by the broad phylogenetic distribution of the gene for the alternative oxidase (AOX) [1, 2]. AOX is a single-subunit enzyme able to bypass respiratory complexes III and IV, transferring electrons from ubiquinone to oxygen without proton pumping and ATP production. It is believed to buffer stresses affecting oxidative phosphorylation, whether due to overload, environmental insults or genetic damage. However, AOX, as well as other alternative respiratory enzymes, was lost independently during the early evolution of both vertebrates and arthropods. This raises the question of what disadvantages AOX might have conferred on the survival and/or reproductive fitness of the ancestors of these taxa.

In fact, only marine invertebrates appear to still possess genes for alternative enzymes [1–3]. Nonetheless, when the AOX gene from the tunicate *Ciona intestinalis* (Ascidiacea) was expressed in cultured human cells, it promoted resistance to inhibitors of complexes III and IV [4] and compensated for the growth defects and oxidant-sensitivity of complex IV-deficient cell-lines [5]. In the fruitfly *Drosophila melanogaster*, transgenic expression of *C. intestinalis* AOX had no apparent effect on viability, reproduction and health of the organism [6]. It conferred resistance to otherwise lethal levels of complexes III and IV inhibitors [6], mitigated locomotor defects in the *dj-1β* Parkinson’s disease model [6] and ameliorated diverse phenotypes associated with complex IV deficiency [7], defective mtDNA replication [8], or expression of human β-amyloid [9]. *C. intestinalis* AOX has also been successfully expressed in the mouse without any significant effect on major physiological parameters [10, 11]. It led to decreased production of reactive oxygen species (ROS) when the respiratory chain was blocked, was able to support cyanide-resistant respiration by intact organs, and conferred prolonged protection against lethal concentrations of hydrogen cyanide in whole animals [10, 11].

These benefits of AOX expression raise the possibility of its eventual use for treatment of human patients with complexes III and/or IV deficiencies, as a by-pass therapy [12]. However, the advantages of AOX expression in ‘higher’ animal systems under pathological conditions contrasts with its evolutionary loss from the genome of vertebrates and arthropods. A detailed study of the biology of the alternative pathways is therefore required before any use of this enzyme in gene therapy for mitochondrial or neurodegenerative disorders can be contemplated [13].

We set out to investigate the paradox between the beneficial by-pass and the possible maladaptive consequences of AOX expression in higher metazoans, by directly testing its effects on male reproductive function. Sperm are highly dependent on biological energy. Therefore, we reasoned that it may be a relevant target in which to test for subtle but functionally meaningful detriments of AOX expression. We challenged *D. melanogaster* males expressing AOX in competition assays with AOX-nonexpressors. AOX produced a clear impairment to reproductive function that was associated with decreased production of mature sperm cells. This suggests that possible gene therapy applications of AOX must be developed with caution.

## Results

### Males expressing AOX are defective in sperm-competition assays

In *Drosophila*, as in many species, females typically mate with a succession of males, storing sperm inside the female body to fertilize oocytes as they mature [14]. Sperm competition occurs by means of a substitution mechanism, in which a second male dislodges the sperm of the first male from the female’s reproductive tract and replaces it with its own gametes [14, 15]; this is apparently the motive force for the correlative evolution between the male’s giant sperm cells and the female’s spacious sperm-storage organs [16]. Newly acquired sperm competes with and usually displaces that from previous males, unless it is functionally compromised. These mechanisms can drive evolution by maximizing male reproductive success, such as by the development of larger testes, production of more abundant, more viable or longer-lasting gametes, or by modifying the response to elevated competition risk, among other characteristics [17].

To test the reproductive success of *D. melanogaster* males expressing AOX, we performed sperm-competition assays between flies carrying three copies of the *α-tubulin-AOX* construct, as described previously (3X*tub-AOX* line: genotype *tub-AOX* ^*35*^/Y; *tub-AOX* ^*112*^/*tub-AOX* ^*112*^; *tub-AOX* ^*7*^/*tub-AOX* ^*7*^ [7]) and wild-type males of the same genetic background (*w* ^*1118*^). In the ‘defensive’ approach, 3X*tub-AOX* males were first allowed to mate with virgin *w* ^*1118*^ females, which were then mated with *w* ^*1118*^ males in the absence of 3X*tub-AOX* males. Progeny from the competing males can be distinguished by eye-colour: those from transgenic males have red eyes, whilst those from *w* ^*1118*^ males have white eyes. Controls consistently show that the eye-colour marker, as such, has no influence over the outcome (Additional file 1: Fig. S1).

**Fig. 1:**
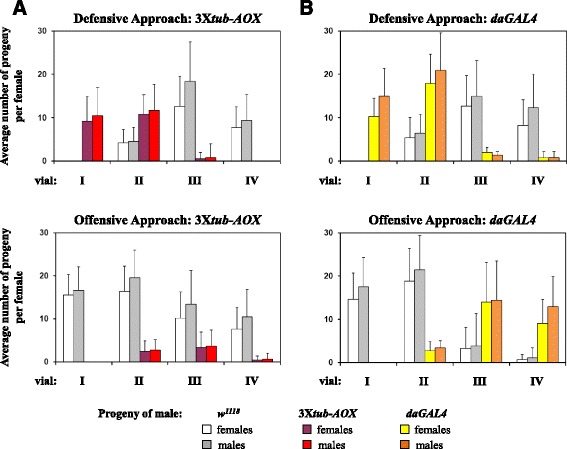
AOX-expressing males are defective in sperm-competition assays. In the defensive paradigm (*upper panels*), virgin *w* ^*1118*^ females (white eyes) were initially crossed individually with transgenic males (red eyes), and then with *w* ^*1118*^ males (white eyes). In the offensive paradigm (*lower panels*), the virgin *w* ^*1118*^ females were first crossed with *w* ^*1118*^ males, and then with transgenic males (see Methods for details). The transgenic males used were 3X*tub-AOX* (*tubAOX* ^*35*^/Y; *tubAOX* ^*112*^/*tubAOX* ^*112*^; *tubAOX* ^*7*^/*tubAOX* ^*7*^) in **a**, and *daGAL4* (X/Y; 2/2; *daGAL4/daGAL4*) in **b**. Vials I-IV represent the mean number of white- or red-eyed progeny ± SD (error bars), eclosed after 3 days from the initial crosses (vial I), 3 days of egg-laying during second crosses (vial II), 3 days of oviposition only, subsequent to second crosses (vial III, males discarded), followed by 5 days of further oviposition (vial IV)

The number of progeny originating from the sperm of 3X*tub-AOX* males was decreased after the females were mated subsequently with *w* ^*1118*^ males (Fig. 1a, *upper panel*). This result is consistent with previously published data using diverse genetic backgrounds [14, 18–21] and with our own data using several other transgenes (Fig. 1b, Additional file 1: Fig. S1–S3): sperm of the first male is replaced (partially or completely) by the sperm of the second male. To implement a rigorous statistical analysis of the findings, we derived the parameter *P1’* which measures the proportion of progeny sired by the first male to mate in sperm-competition assays [22]. This was similar for all lines analyzed here, although 3X*tub-AOX* males sired statistically fewer offspring than some of the AOX-nonexpressor controls (Additional file 1: Table S1).

In contrast, when 3X*tub-AOX* males were challenged in an ‘offensive’ approach, i.e., when the virgin *w* ^*1118*^ females were first crossed with *w* ^*1118*^ males, then with AOX-expressing males, the number of 3X*tub-AOX* progeny never overcame the number of *w* ^*1118*^ progeny (Fig. 1a, *lower panel*). In fact, even after 10 days since *w* ^*1118*^ males were removed from the mating vials, eggs fertilized with the original male sperm were still preferentially laid over the ones fertilized with 3X*tub-AOX* sperm (Fig. 1a, *lower panel*, vial IV). Control experiments performed with red-eyed *daughterless-GAL4* (*daGAL4*) (Fig. 1b, *lower panel*) and *tubulin-GeneSwitch* (*tubGS*) and also *UAS-empty* ^*2nd*^ and *UAS-empty* ^*3rd*^ (Additional file 1: Fig. S1) males imply that the failure of AOX-expressing males to compete successfully in the offensive paradigm is specific to AOX, and is not a property of transgenic lines in general. The defect appeared to be AOX transgene-dose dependent. Males carrying only two copies of the *tub-AOX* transgene (Additional file 1: Fig. S2) appeared less impaired in the ‘offensive’ paradigm than those carrying three (Fig. 1a), whilst those with just a single copy of *tub-AOX* were not significantly different from control males (Additional file 1: Fig. S2). These conclusions reflect the statistical analysis of the proportion of progeny sired by the second male (*P2’*, [22]), wherein 3X and 2X*tub-AOX* males were significantly impaired in the offensive paradigm compared to all control classes (Additional file 1: Table S2), although the differences between 3X and 2X*tub-AOX* (Additional file 1: Table S2) themselves were not significant. The observed phenotypes also correlate approximately with the amount of expression of the *tub-AOX* transgene in the male reproductive system, observed by immunoblotting (Additional file 1: Fig. S4).

However, expressing GAL4-dependent *UAS-AOX* using the *daGAL4* driver [6], produced no loss of sperm-competitiveness (Additional file 1: Fig. S3A, and Table S2), despite the fact that the level of AOX protein in the male reproductive system was comparable with that expressed from multiple copies of *tubAOX* (Additional file 1: Fig. S4). These data suggest that the effect of AOX on sperm competitiveness depends on the precise cellular context of its expression rather than simply its overall amount.

### AOX-expressing males accumulate a decreased amount of mature sperm

**Fig. 2:**
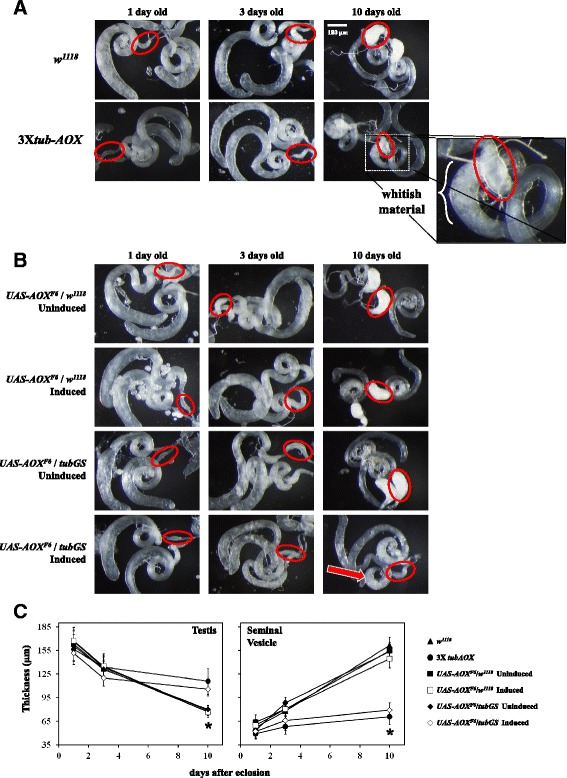
Mature sperm cells do not accumulate in the seminal vesicles (SVs) of AOX-expressing males. Representative samples of dissected testes and SVs from adult males of the indicated age are shown for control and AOX-expressing lines: **a** *w* ^*1118*^ (X/Y; 2/2; 3/3) and 3X*tub-AOX* (*tubAOX* ^*35*^/Y; *tubAOX* ^*112*^/*tubAOX* ^*112*^; *tubAOX* ^*7*^/*tubAOX* ^*7*^); **b** *UAS-AOX* ^*F6*^/*w* ^*1118*^ (X/Y; *UAS-AOX* ^*F6*^/2; 3/3) and *UAS-AOX* ^*F6*^/*tubGS* (X/Y; *UAS-AOX* ^*F6*^/2; *tubGS*/3) in the absence (uninduced) or presence (induced) of 200 μM mifepristone. AOX protein induction by mifepristone was visualized by immunoblotting (Additional file 1: Fig. S5). Red circles indicate one of the dissected SVs of the representative samples of the indicated genotype. Note the underdeveloped state of the SVs in 10 day-old flies from the lines expressing AOX either constitutively (3X*tub-AOX*) or inducibly (*UAS-AOX* ^*F6*^/*tubGS* plus mifepristone). For an explanatory visualization of *D. melanogaster* reproductive organs, see Additional file 1: Fig. S6A. In **a**, the *inset* highlights the whitish material that accumulates in the proximal end of the testis in 3X*tub-AOX* males; in **b**, the *red arrow* points to the same material for *UAS-AOX* ^*F6*^/*tubGS* males treated with mifepristone. **c** Quantification of the data shown in **a** and **b**, in which the data points represent the mean thickness of 10–20 dissected organs ± SD (error bars). * indicates statistically significant difference between organs of AOX-expressing and nonexpressing males (*p* ≤ 0.01). Additional file 1: Fig. S8B illustrates schematically the thickness measurement protocol used to estimate the amount of immature (in the testes) and mature (in the SVs) sperm cells per fly

Successful sperm competition in *Drosophila* depends both on the number and quality of spermatozoids and on the compatibility between the male’s seminal fluid proteins and their receptors in the female reproductive tract [17, 23–25]. We checked how the production of mature spermatozoids in the 3X*tub-AOX* males is affected by dissecting the reproductive organs in adult males of increasing age. Spermatogenesis in *D. melanogaster* starts at the distal tip of the testes, taking place inside cysts. As cell differentiation proceeds, the cysts move towards the proximal end of the testes, where these organs connect to the SVs. The mature spermatozoids are then deposited in the SVs, where they are stored until mating [26]. Therefore, as adult males age, the testes tend to get thinner, because differentiation in the cysts proceeds and the fully formed spermatozoids move to the SVs, which in turn increase in size. As shown in Fig. 2 (panels a and c), the thickness of the testes of control flies decreases >50% in the first 10 days of adult life, whereas the SVs triple in size. In contrast, the testes of 3X*tub-AOX* males exhibit a much less pronounced decrease in thickness (~25%), whilst at the same time their SVs remain immature. In addition, we observed an accumulation of whitish material at the proximal end of the testes of 10-day-old 3X*tub-AOX* males (Fig. 2a, *inset*), which we investigated further (see below). The mature spermatozoids produced by these males, although few in number, did not appear to have any motility defects, as judged by visual inspection.

We were able to produce a similar phenotype using the inducible GeneSwitch (*tubGS*) system, in which the same *α-tubulin* promoter as used in the *tub-AOX* constructs controls the expression of a modified GAL4 that is inducible by mifepristone (RU486). This allows time-regulated expression of a GAL4-dependent AOX transgene in exactly the same tissues as in the *tubAOX* lines. By transferring males to food-vials containing the inducing drug on the day of eclosion, any possible developmental disturbance is avoided, whilst accurate controls can be implemented, notably flies with transgene, but lacking driver and/or drug (see Additional file 1: Fig. S5 for immunoblots indicating tight regulation of AOX protein levels using this system). Similarly as for 3X*tub-AOX* males, the testes of *UAS-AOX* transgenic males driven by *tubGS* in the presence of mifepristone remained thick during the first 10 days of adult life, whilst their SVs remained small (Fig. 2b and c). These organs were as wild-type in the absence of the driver or inducing drug (Fig. 2b and c). The normal thinning of the testis over the first 10 days of adult life was also seen when both the driver and inducing drug were present, whether driving a control transgene, GFP, a catalytically inactive form of AOX, mutAOX [27] or even with no transgene at all (Additional file 1: Fig. S6A, S6C). In these cases, however, the SV remained undeveloped, but only when driver and drug were both present (Additional file 1: Fig. S6B, S6D). The whitish material near the proximal end of the testis also accumulated prominently when *tubGS* was used to express AOX (but not GFP or mutAOX), and again only under inducing conditions (Fig. 2b, *red arrow*). To confirm that this is an *α-tubulin* promoter-specific phenotype, we dissected the reproductive organs of 10-day-old males expressing *UAS-AOX* driven by *daGAL4*. The SVs had a normal morphology (Additional file 1: Fig. S7), and appeared to have accumulated a similar amount of mature sperm cells as controls, in agreement with the results of the sperm-competition assays (Additional file 1: Fig. S3).

Other reproductive organs were also evaluated morphologically (see Additional file 1: Fig. S8A for schematics of the reproductive system of *D. melanogaster* males), but no obvious alterations were observed between AOX-expressing and control males, based on visual inspection of >70 dissected flies of each genotype. Although AOX was not expressed in the accessory glands (Additional file 1: Fig. S9A), we also checked the presence of Sex Peptide in these organs by immunostaining and confocal microscopy, and again observed no difference (Additional file 1: Fig. S9B). Sex Peptide is one of the most important components of the male’s seminal fluid; it has been implicated in reproductive success in sperm-competition assays [24, 28] and its action is dependent on the compatibility with receptors in the female reproductive tract [23]. Altogether, our data indicate that AOX-expressing males are defective in sperm-competition assays due to a decreased production of mature spermatozoids.

### AOX is expressed in cells of the testis and seminal vesicle sheaths

Although the SVs of AOX-expressing males present perhaps the most pronounced phenotype in our study, these are purely storage organs for mature sperm cells [26], and their morphology was not diagnostic for AOX expression (Additional file 1: Fig. S6B).

**Fig. 3:**
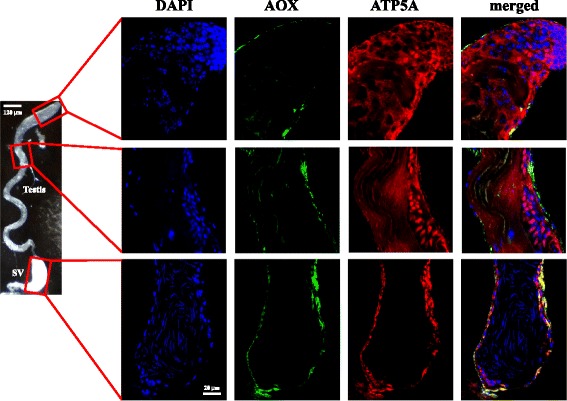
AOX protein is localized in mitochondria of cells from the testis and seminal vesicle (SV) sheaths. Immunoflorescence staining and confocal microscopy for the indicated proteins and DAPI, from a 10-day old male of genotype X/Y; *UAS-AOX* ^*F6*^/2; *tubGS*/3, in the presence of mifepristone. AOX protein is localized to the outermost cell layer of these organs, where it overlaps that for the mitochondrial marker ATP5A, but not to germline cells at any stage of differentiation. The light microscopy image (left) is from a control male (*w* ^*1118*^), used for illustration purpose only. Spermatogenesis takes place inside cysts, starting at the tip of the testes (*upper panels* - note the concentration of nuclei that represent the first stages of this process) and moving towards the proximal end of the organ as cell differentiation proceeds (*middle panels* - note the presence of a cyst containing onion-stage spermatids with their single mitochondrial mass, called the Nebenkern). At the proximal end of the testis, mature spermatozoid cells are released from the cyst and stored in the SV (*lower panels* - note the needle-shaped nuclei that is typical of these cells). Note also the low-level AOX expression in the somatic cells of the cysts, but not in the germline. See Additional file 1: Fig. S10 for comparable images from *3Xtub-AOX* males

Therefore, we hypothesized that the defect in sperm production caused by AOX expression most likely affects the testes, where spermatogenesis takes place. The *α-tubulin* promoter has been used previously to drive transgene expression in the fly testis in both somatic cells and the germline (from the stem-cell stage to late spermatocytes, reviewed in [29]). However, using immunofluorescent confocal microscopy (Fig. 3 and Additional file 1: Fig. S10), AOX expression from the *α-tubulin* promoter, whether directly (Additional file 1: Fig. S10) or driven by *tubGS* plus mifepristone (Fig. 3), was below the level of detection in germline cells, and seen only at very low levels in some somatic cyst cells. Nevertheless, it was abundantly expressed in the somatic cells of the testis and SV sheaths. In addition, all of the immature stages of cell differentiation appeared to be present (Additional file 1: Fig. S11), judging by the characteristic changes in nuclear morphology, mitochondrial network arrangement and cellular elongation that the germ line cells go through towards the final steps in spermatogenesis [26].

At higher resolution, it was clear that not all cells in the ensheathing tissues of the testes and SVs express AOX at a high level when driven by the *α-tubulin* promoter (either directly, as 3X*tub-AOX* or indirectly, through *tubGS*). Some cells highly expressing ATP5A, a complex V subunit traditionally used as a mitochondrial marker, appeared negative for AOX (Additional file 1: Fig. S12A). Using *UAS-StingerGFP* in combination with the *tubGS* driver, in order to mark the positive cells with GFP in the nucleus, we observed high expression in specific cells in the outermost sheath-cell layer (Additional file 1: Fig. S12B). The inner sheath-cell layer, formed of smooth-muscle cells with multiple small nuclei, appeared negative for AOX or GFP, in agreement with co-staining for AOX and actin (Additional file 1: Fig. S13A) in the testes of 3X*tub-AOX* males. Positive cells most likely correspond with the pigment cells, which carry a single large nucleus, and are abundant around the SVs and the proximal end of the testis, where these two organs connect [30]. This is supported (Additional file 1: Fig. S13B–D) by co-staining for the *empty spiracles* gene product (ems), which is a marker for the pigment cells [31, 32]. Partial three-dimensional reconstruction of the microscopy images shows that the AOX and ems signals are located in the outermost cell layer of the sheath of the SV (Additional file 1: Fig. S13C) and testis (Additional file 1: Fig. S13D), whilst the underlying (smooth muscle) cells stain highly for ATP5A and actin. Note that the pigment cells are abundant in the anatomical region where the most pronounced morphological alterations were found in AOX-expressing males, marked by the accumulation of whitish material at the proximal end of the testis combined with a decreased amount of mature sperm cells in the SVs (Fig. 2a).

### Mature-looking spermatozoids are lodged in the proximal end of the testis in AOX-expressing males

The proximal end of the *D. melanogaster* testis is also the region where the individualization process starts. This process is essential for the final stage of elongated spermatid differentiation [26], and is accomplished by an individualization complex (IC) that is assembled in the cyst region containing the spermatid nuclei and which then moves towards the tip of the tails, collecting syncitial cytoplasm and creating cystic bulges and waste bags (WBs) along the way [33, 34]. By staining the testis samples for actin and activated caspase-3, two components of the ICs, we observed no significant differences in the number of starting and established ICs in males expressing AOX driven by *tubGS*, but did observe an elevated number of WBs (Additional file 1: Fig. S14). Most noticeably, we observed in these males a dramatic change in the distribution of ICs throughout the organ. Starting ICs are usually present in the proximal region of the testis, but in 10 day-old males expressing AOX driven by *tubGS*, a significant number of them were found towards the middle region of the organ. Established ICs, which are usually found all along the testes, were concentrated at the middle and proximal end of the AOX-expressing testes. Finally, WBs appeared to be distributed in all testis regions in the AOX-expressing males, whereas in control testes they accumulated at the distal end (Additional file 1: Fig. S14C). Our observations imply that AOX expression driven by *tubGS* in the somatic cells of the cysts, and/or in the pigment cells of the testis sheath causes internal rearrangements in this organ, disrupting the production and delivery of mature sperm.

**Fig. 4:**
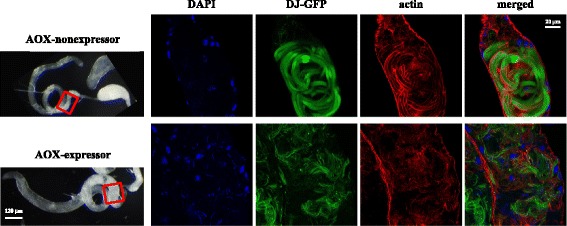
Individualized sperm cells remain lodged in the testes of AOX-expressing males. Fluorescence imaging of testes of 10-day old males of genotype X/Y; *UAS-AOX* ^*F6*^/2; *tubGS*/DJ-GFP, under uninduced (*upper panels*) and mifepristone-induced (*lower panels*) conditions, was performed using phalloidin-TRITC to detect actin and the DJ-GFP fluorescence to detect the tails of elongated spermatids and mature spermatozoids. Note that in the proximal region of the testis (fluorescent confocal images, right, and *red boxes* on the white-light microscopy images, left) of the uninduced control sample (AOX-nonexpressor) only the bundles of not-yet-individualized elongated spermatids are observed, whereas in the same region in AOX-expressing males these cells appear individualized, tangled and/or disorganized, accounting for the accumulated whitish material shown in Fig. 2

To investigate the nature of the whitish material found at the proximal end of the testis of AOX-expressing males, we used a transgenic construct that expresses GFP-tagged Don Juan protein (DJ-GFP) in the tail of elongated spermatids and mature spermatozoids [35, 36], enabling us to evaluate the transition between these two stages. The expression of DJ-GFP revealed a high concentration of what appeared to be individualized mature spermatozoids in the proximal end of the testis of AOX-expressing males (Fig. 4, *lower panels*). In control males, few of these individualized GFP-positive cells were found in this region of the testes (Fig. 4, *upper panels*), as expected, given that they should move to the SVs after individualization. The failure of the individualized, elongated spermatids to move into the SV and the associated disturbance in the spatial distribution of ICs could account for the accumulation of the whitish material seen by light microscopy, and is the simplest explanation for the defect seen in sperm-competition assays, although the reason why they are retained in the testis is not obvious morphologically.

## Discussion

In previous studies [6, 7] we reported that constitutive expression of *C. intestinalis* AOX in *Drosophila* did not affect viability or fertility under non-competitive lab conditions. Here we demonstrate that transgenic AOX expression in *Drosophila* under the *α-tubulin* promoter does cause a clear detriment to male reproductive success, using a stringent sperm-competition assay. This was accompanied by a decreased and disorganized production of mature spermatozoids. Although some sperm cells appear to complete the process of morphological differentiation, following individualization they appear to remain lodged in the proximal region of the testis, in the outer sheath of which AOX is highly expressed.

AOX, when expressed directly under the *α-tubulin* promoter or via the *tubGS* driver, is present at high abundance in the pigment cells of the testis sheath and SV, and is also found at very low levels in some somatic cyst cells. However, it is below the level of detection in the germline. The *α-tubulin* promoter has been reported to drive germline expression of transgenes up to the late spermatocyte phase [29]. Clearly, this does not apply to the construct we used [7]. Surprisingly we also observed no abnormalities during spermatogenesis in the mitochondrial network (Additional file 1: Fig. S11), the reorganization of which provides the structural platform for sperm axoneme formation [37]. On the other hand, the elevated number and altered distribution of WBs in the testes of AOX-expressing males may reflect increased recycling of dysfunctional germ cells, where differentiation has failed. We posit that the specific disturbance in spermatogenesis produced by AOX expression is most likely due to deranged signaling from somatic cells, rather than low-level AOX expression in the germline itself.

The precursor cells of the testis and SV sheaths have two distinct developmental origins: the pigment cells originate in the gonad imaginal disc, along with the testes, whereas the muscle cells and the SVs originate in the genital disc [38, 39]. When the two organs start fusing, migration of pigment and muscle cells proceeds in opposite directions with the gonad and the SVs each acquiring an outer layer of pigment cells and an inner layer of muscle [30]. The failure to produce pigment cells during development is known to lead to aberrant muscle-cell migration, causing abnormal testis morphology and male sterility, without affecting the germline or somatic cell-types that support spermatogenesis [30]. This interdependent migration implies the existence of a signaling network between the pigment and the muscle cells. Our findings suggest that communication between these cell-types might continue after testis development is complete. We suggest that AOX expression in the pigment cells might in some way disrupt their ability to deliver signals required for the completion of spermatogenesis, and/or to signal muscle contraction needed to guide individualized mature spermatozoids from the proximal end of the testis into the SV, leading to the accumulation of cysts in late stages of maturation throughout the testis. This is supported by the fact that a low percentage of testes of 3X*tub-AOX* males (~5% of total testes analyzed, Additional file 1: Fig. S15) presented malformations very similar to those reported for males unable to develop pigment cells [30]. Although the majority of testes are morphologically normal in most 3X*tub-AOX* males at eclosion, they remain thick over the first 10 days of adult life (Fig. 2B), indicating a functional defect in sperm production and delivery that would be sufficient to account for the decreased reproductive success under competitive conditions (Fig. 1). To prove that AOX expression in the sheath pigment cells was the cause of the observed sperm and reproductive defects would require a driver that enables its expression specifically in these cells. Since no such driver is currently available, we must rely instead on the strongly suggestive correlations we have documented here.

The effect of AOX on cell-cell communication could result from one of several metabolic disturbances conferred by the enzyme. If constitutively activated by local metabolic conditions in specific cells, AOX could participate significantly in respiratory electron flow, impairing ATP production or other downstream processes dependent on respiratory energy, such as the buffering of calcium (reviewed in [40]). A calcium signaling pathway has been implicated in the upregulation of Wnt-2 [41], which regulates differentiation in many cellular contexts, including the testis pigment cells during *Drosophila* development, promoting muscle cell migration and attachment [30]. *Drosophila* males mutant for Wnt-2 are sterile due to severe testis malformations [30], whereas males expressing AOX are fertile and appear morphologically normal at eclosion, with a functional defect only revealed by a stringent competition assay and by the apparent retention of sperm within the testis during the first 10 days of adult life.

An interference with cell signaling may also involve ROS. AOX has been reported to decrease mitochondrial ROS production even under conditions of normal oxidative phosphorylation activity [6, 42]. In either case, the very subtle phenotype, with all other physiological and developmental functions apparently unaffected, presents a challenge for understanding the mechanism.

Clearly, much remains to be learned about the physiological effects of AOX expression. Our findings indicate the importance of studying these effects and their mechanisms in detail, before implementing AOX in gene therapy. Some of its properties may need to be modified, before the enzyme can be safely deployed.

## Conclusions

AOX offers a potential therapeutic strategy for buffering pathological stresses in mitochondria. Whilst it is, indeed, able to compensate for a number of pathological insults, it has remarkably little, if any effect on normal physiology of model organisms, including *Drosophila*. Transgenic flies ubiquitously expressing AOX were earlier found to exhibit normal development, reproduction and lifespan.

In the present study we took this analysis to a deeper level, by applying a more stringent test of reproductive capacity, the well-established sperm-competition assay. Despite being fertile, AOX-expressing males showed a specific detriment in this test, which correlates with total transgene expression dose. Morphologically, we found this to be correlated with spatially deranged spermatogenesis and failure to release mature sperm into the storage organ (seminal vesicle) in normal quantities, although the defect is inferred to arise in the somatic pigment cells of the testis, not the germline. This is the first demonstration of a developmental defect caused by AOX expression in a metazoan, and needs also to be taken into account when considering therapeutic uses of AOX in humans.

## Methods

### Fly stocks and maintenance

Standard lines *w* ^*1118*^, CyO and TM3,Sb balancers, *UAS-StingerGFP*, *daGAL4*, *tubGS*, and DJ-GFP were obtained from stock centres. The lines *UAS-AOX* ^*F6*^ [6], *tub-AOX* ^*35*^, *tub-AOX* ^*112*^, *tub-AOX* ^*7*^, 2X*tub-AOX*, 3X*tub-AOX* [7], *UAS-AOXwt* ^*8.1*^, *UAS-AOXmut*, *UAS-empty* ^*2nd*^, and *UAS-empty* ^*3rd*^ [27] were described previously. All fly lines were backcrossed into the *w* ^*1118*^ background for six-to-ten generations, and were maintained in standard diet at 25 °C [6]. RU486 (mifepristone, Sigma, USA) was added to the diet at the indicated concentrations for induction of transgene expression using the *tubGS* line.

### Sperm-competition assays

Sperm-competition assays were conducted using two approaches. In the defensive paradigm, approximately 50 virgin *w* ^*1118*^ females (white eyes), aged between 3 and 7 days, were initially allowed to mate individually with 5–8 day-old (virgin) transgenic males (red eyes, *w+*) for 3 days. The males were discarded and the females transferred to new vials and allowed to mate individually with 5–8 day-old *w* ^*1118*^ males (white eyes), for a further 3 days, after which the second males were also discarded. The females were again transferred to new vials, allowed to lay eggs for 3 days, then transferred finally for further egg laying over 5 days. In the offensive paradigm, approximately 50 virgin *w* ^*1118*^ females (white eyes) were first crossed with *w* ^*1118*^ males (white eyes), and then with transgenic males (red eyes, *w+*), following the same mating and egg laying scheme of the defensive paradigm. Reproductive success was measured by counting the number of red- and white-eyed progeny, which represent, respectively, the progeny of transgenic and *w* ^*1118*^ males. The small number of vials derived from any single female that contained progeny exclusively of one eye colour were considered to represent only a single mating and were excluded from the analysis. Data were plotted as mean number of progeny per female of each eye colour, in the four successive mating/egg laying vial sets, ± standard deviation.

### Dissection and imaging of male reproductive organs

Reproductive organs were dissected by anesthetizing males of the indicated age and genotype with CO_2_ prior to transferring to a dissection board containing phosphate-buffered saline (PBS). Using thin dissection forceps, the internal organs were removed from the abdomen by dislocating the external genitalia, followed by manual isolation of the reproductive organs, which were imaged immediately using the Nikon SMZ 745 T system under white light. The images were analyzed and testis and SV thickness was measured using NIS Elements D4.20 software (Nikon Instruments Software, Netherlands).

For immunofluorescence imaging, dissected organs were fixed in 4% paraformaldehyde for 20 min at room temperature, and then transferred to PBS prior to permeabilization. The organs were rinsed twice with PBS containing 0.1% Triton X-100 and 0.1% BSA, then permeabilized and blocked with PBS containing 1% Triton X-100 and 1% BSA for 30 min at room temperature, followed by overnight incubation at 4 °C with the primary antibody diluted in PBS containing 0.3% Triton X-100 and 0.5% BSA. The samples were then rinsed twice and washed three times (30 min each) with PBS containing 0.1% Triton X-100 and 0.1% BSA at room temperature, followed by overnight incubation at 4 °C with the secondary antibody diluted in PBS containing 0.3% Triton X-100 and 0.5% BSA. Washes were performed as described above, and samples were rinsed with PBS and then Milli-Q water, prior to transferring to optically clear 35 mm glass bottom dishes (MatTek, USA) containing ProLong Gold Antifade Mounting medium with DAPI (Molecular Probes, Life Technologies). Primary antibodies used were rabbit polyclonal anti-AOX (1:10,000, [6]), mouse monoclonal anti-ATP5A (1:1000, Abcam, UK), rabbit polyclonal anti-activated caspase-3 (1:200, Cell Signaling Technology, USA), rabbit polyclonal anti-Sex Peptide (1:300, gift from Dr. Shanjun Chen) and rabbit polyclonal anti-empty spiracles (1:400, Antibody Verify, USA). Fluorescent secondary antibodies were Alexa 488-conjugated goat anti-rabbit IgG (1:1000) and Alexa 568-conjugated gat anti-mouse IgG (1:1000) (Fisher Scientific, USA). Actin was stained using phalloidin-TRITC (1 μg/ml, Sigma, USA). Samples were imaged using an Andor Spinning Disc or Zeiss LSM 780 laser-scanning confocal microscope, respectively with Andor iQ 3.0 or ZEN 2011 SP3 (black edition) software. Images were analyzed with ImageJ software (National Institutes of Health, USA). No signals were observed when the primary antibodies were omitted.

### Immunoblotting

Protein extracts from whole adult males (20–30) and from dissected testes and SVs of 50–100 males were prepared by gentle grinding of tissues in 50–200 μl PBS containing 1.5% Triton X-100 and Complete, Mini, EDTA-free Protease Inhibitor Cocktail (Roche, Switzerland). The suspension was centrifuged at 16,000 *g*_*max*_ for 10 min at 4 °C, and supernatant protein concentration was measured by the Bradford method. Crude mitochondrial preparations were obtained by homogenizing 50–100 adult males in ice-cold isolation buffer (250 mM sucrose, 5 mM Tris, 2 mM EGTA, pH 7.4) followed by centrifugation at 200 *g*_*max*_ for 3 min at 4 °C. The supernatant was transferred to a new tube and centrifuged at 9000 *g*_*max*_ for 10 min at 4 °C. The pellet was resuspended in 50 μl of isolation buffer, and protein concentrations were determined using the Bradford method.

100 μg of total protein extracts and 40 μg of mitochondrial preparations were mixed with 5× Laemmli buffer (10% SDS, 50% glycerol, 25% 2-mercaptoethanol, 0.02% bromophenol blue and 0.3125 M Tris-HCl, pH 6.8), denatured at 95 °C for 5 min and resolved by SDS-PAGE on 4–20% or Any kD Criterion Precast Gels (Bio-Rad) at 120 V for approximately 1.5 h. Proteins were transferred to nitrocellulose membranes using the iBlot Gel Transfer system (Invitrogen, USA) for 7 min at room temperature. Membranes were blocked in TBST (0.15 M NaCl, 50 mM Tris-HCl, 0.05% Tween 20, pH 7.6) containing 5% dried nonfat milk for 2 h at room temperature or overnight at 4 °C. Primary antibodies (rabbit polyclonal anti-AOX [1:10,000, [6]], mouse monoclonal anti-ATP5A [1:5000, Abcam, UK], mouse monoclonal anti-PDH E1α [1:5000, Abcam, UK], and mouse monoclonal anti-GAPDH [1:5000, Abcam, UK]) were incubated for at least 1 h at room temperature, followed by three washes (10 min each) with TBST. Secondary antibodies (HRP-conjugated goat anti-rabbit and anti-mouse IgGs [1:10,000, Bio-Rad, USA]) were incubated for at least 2 h at room temperature, and washed as described. Membranes were incubated in Luminol substrate detection system Immun-Star HRP (Bio-Rad, USA), and chemiluminescence signals were detected on Fuji Medical X-ray films (Fujifilm, Japan). Band densitometry was performed with ImageJ software (National Institutes of Health, USA).

### Statistical analyses

Measurements of organ thickness were performed as schematized in Additional file 1: Fig. S8B and tested for statistical differences among the indicated groups of fly males using one-way ANOVA with Newman-Keuls post hoc test. For analysis of sperm-competition assays, the parameters *P1’* and *P2’* [22] were tested for statistical differences among the indicated groups of fly males, using one-way ANOVA with Tukey post hoc test. The analyses were performed using Prism software, version 5 (GraphPad Software, USA).

## Additional file

Additional file 1: Table S1. Proportion of progeny sired by the red-eyed male when this is the first male to mate in sperm competition assays against *w* ^*1118*^ males (*P1*´). **Table S2.** Proportion of progeny sired by the red-eyed male when this is the second male to mate in sperm competition assays against *w* ^*1118*^ males (*P2*´). **Figure S1.** The sperm of eye-color control and *tubGS* males shows normal competitive properties. **Figure S2.** The defect in sperm-competition assays is AOX dose-dependent. **Figure S3.** AOX expressed under the *daughterless* promoter does not decrease sperm competitiveness. **Figure S4.** AOX protein levels in testes and seminal vesicles of diverse AOX-expressing lines. **Figure S5.** Induction of AOX expression using the *UAS-AOX* ^*F6*^/*tubGS* system. **Figure S6.** Altered testis morphology during adulthood is diagnostic for AOX expression. **Figure S7.** Mature sperm cells accumulate normally in the seminal vesicles (SVs) of males expressing AOX under the control of the *daGAL4* driver. **Figure S8.** Schematic diagrams and light microscopy of *D. melanogaster* male reproductive organs [43]. **Figure S9.** AOX is not present in the accessory glands and does not interfere with Sex Peptide production. **Figure S10.** AOX protein is localized in mitochondria of cells from the testis and seminal vesicle (SV) sheaths in *3Xtub-AOX* males. **Figure S11.** Nuclear and mitochondrial morphologies in germline cells are not altered in *3Xtub-AOX* males. **Figure S12.** AOX is expressed in the outermost cell layer of the testis and *SV* sheaths. **Figure S13.** AOX localizes to the pigment cell layer of the testis and SV sheaths of *3Xtub-AOX* males [44]. **Figure S14**. AOX-expressing males present alterations in sub-testicular structures. **Figure S15.** Testis malformation in *3Xtub-AOX* males. (PDF 3255 kb)

## Abbreviations

AOX: Alternative Oxidase
ATP: Adenosine Triphosphate
GFP: Green Fluorescent Protein
IC: Individualization Complex
ROS: Reactive Oxygen Species
SV: Seminal Vesicles
UAS: Upstream Activating Sequence
