# Supplementary material for: Expression of Ciona intestinalis AOX causes male reproductive defects in Drosophila melanogaster
Source: BMC Dev Biol. 2017 Jul 3;17:9. doi: 10.1186/s12861-017-0151-3 (PMC5496232; doi:10.1186/s12861-017-0151-3)

Expression of *Ciona intestinalis* AOX causes male reproductive defects in *Drosophila melanogaster*

Sina Saari*, Ana Andjelković*, Geovana S. Garcia, Howard T. Jacobs and Marcos T. Oliveira

SUPPLEMENTARY INFORMATION

Table S1. Proportion of progeny sired by the red-eyed male when this is the first male to mate in sperm competition assays against w^{1118} males (*P1´*).

Genotype	mean *P1´*	standard deviation	statistical class*
3X*tubAOX*	0.276	0.155	a
2X*tubAOX*	0.326	0.158	a,b
$tubAOX^{7}$	0.491	0.199	a,b
$tubAOX^{112}$	0.436	0.182	a,b
daGAL4	0.286	0.093	a,b
$UAS\text{-}empty^{2nd}$	0.526	0.187	b
$UAS\text{-}empty^{3rd}$	0.612	0.200	b
tubGS	0.595	0.060	b
$UAS\text{-}AOX^{F6}/daGAL4$	0.366	0.199	a,b
UAS-GFP/daGAL4	0.491	0.240	b

* $p < 0.05$, according to a one-way ANOVA, followed by the Tukey *post-hoc* test. Note that *P1´* of 3X*tubAOX* is statistically lower than that of $UAS\text{-}empty^{2nd}$, $UAS\text{-}empty^{3rd}$, *tubGS* and *UAS-GFP/daGAL4*, but is not different than that of any AOX-expressing line and *daGAL4*.

Table S2. Proportion of progeny sired by the red-eyed male when this is the second male to mate in sperm competition assays against w^{1118} males (*P2´*).

Genotype	mean *P2´*	standard deviation	statistical class*
3X*tubAOX*	0.146	0.129	a
2X*tubAOX*	0.234	0.197	a
tubAOX^{7}	0.405	0.193	b
tubAOX^{112}	0.425	0.187	b,c
daGAL4	0.481	0.179	b,c
UAS-empty^{2nd}	0.397	0.176	b,c
UAS-empty^{3rd}	0.653	0.208	d
tubGS	0.399	0.198	b
UAS-AOX^{F6}*/daGAL4*	0.623	0.094	c,d
UAS-GFP/daGAL4	0.670	0.125	c,d

* $p < 0.05$, according to a one-way ANOVA, followed by the Tukey *post-hoc* test. Note that *P2´* of 3X*tubAOX* and 2X*tubAOX* is statistically lower than that of any other line, and that *P2´* of *tubAOX*^{7} is lower than that of the controls *UAS-empty*^{3rd}, *UAS-AOX*^{F6}*/daGAL4* and *UAS-GFP/daGAL4*, although *tubAOX*^{112} has a *P2´* only statistically lower than that of *UAS-empty*^{3rd}.

Supplementary Figure Legends

Figure S1. The sperm of eye-color control and *tubGS* males shows normal competitive properties. Sperm-competition experiments using the defensive and offensive paradigms (*upper* and *lower panels,* respectively) were performed as described in Materials and Methods and in the legend to Figure 1. The transgenic males used were $UAS\text{-}empty^{2nd}$ (X/Y; $UAS\text{-}empty^{2nd}$/ $UAS\text{-}empty^{2nd}$; 3/3, which contain the *mini-white* gene and the *UAS* promoter fused to no transgene, inserted on chromosome 2 [27]) in **A**, $UAS\text{-}empty^{3rd}$ (X/Y; 2/2; $UAS\text{-}empty^{3rd}$/ $UAS\text{-}empty^{3rd}$; 3/3, which contain the *mini-white* gene and the *UAS* promoter fused to no transgene, inserted on chromosome 3 [27]) in **B**, and *tubulin-GeneSwitch* (X/Y; 2/2; *tubulin-GeneSwitch*/*tubulin-GeneSwitch*, expressing the GeneSwitch transcription factor under the same promoter as used for AOX in the 3X*tub-AOX* line) in **C**. Vials I-III here represent the mean number of white- or red-eyed progeny ± SD (error bars), eclosed after 3 days of the initial mating (vial I), 3 days from the second mating (vial II), or the following 7 days of oviposition only (vial III, males discarded).

Figure S2. The defect in sperm-competition assays is AOX dose-dependent. Sperm-competition experiments using the defensive and offensive paradigms (*upper* and *lower panels,* respectively) were performed as described in the Materials and Methods and in the legend to Figure 1. The transgenic males used here were $tubAOX^{112}$ (X/Y; $tubAOX^{112}$/$tubAOX^{112}$; 3/3) in **A**, $tubAOX^{7}$ (X/Y; 2/2; $tubAOX^{7}$/$tubAOX^{7}$) in **B**, and 2X*tub-AOX* (X/Y; $tubAOX^{112}$/$tubAOX^{112}$; $tubAOX^{7}$/$tubAOX^{7}$) in **C**. Vials I-IV represent the mean number of white- or red-eyed progeny ± SD (error bars) according to the scheme described in the legend to Figure 1.

Figure S3. AOX expressed under the *daughterless* promoter does not decrease sperm competitiveness. Sperm-competition experiments using the defensive and offensive paradigms (*upper* and *lower panels*, respectively) were performed as described in the Materials and Methods and in the legend to Figure 1. The transgenic males used here were *UAS-AOX*^{F6}/*daGAL4* (X/Y; *UAS-AOX*^{F6}/CyO; *daGAL4*/TM3,Sb) in **A**, and *UAS-StingerGFP*/*daGAL4* (X/Y; *UAS-StingerGFP*/CyO; *daGAL4*/TM3,Sb) in **B**. Vials I-IV represent the mean number of white- or red-eyed progeny ± SD (error bars), according to the scheme described in the legend to Figure 1.

Figure S4. AOX protein levels in testes and seminal vesicles of diverse AOX-expressing lines. Total protein extracts from whole adult males and from dissected testes and seminal vesicles were prepared and immunoblotted as described in Materials and Methods (*left hand panels*). The AOX-expressing lines used were: *tubAOX*^{112} (X/Y; *tubAOX*^{112}/*tubAOX*^{112}; 3/3), *tubAOX*^{35} (*tubAOX*^{35}/Y; 2/2; 3/3), *tubAOX*^{7} (X/Y; 2/2; *tubAOX*^{7}/*tubAOX*^{7}), 3X*tub-AOX* (*tubAOX*^{35}/Y; *tubAOX*^{112}/*tubAOX*^{112}; *tubAOX*^{7}/*tubAOX*^{7}), and *UAS-AOX*^{F6}/*daGAL4* (X/Y; *UAS-AOX*^{F6}/2; *daGAL4*/3). The control lines used were: *UAS-AOX*^{F6}/*w*^{1118} (X/Y; *UAS-AOX*^{F6}/2; 3/3), *UAS-AOX*^{F6}/*UAS-AOX*^{F6} (X/Y; *UAS-AOX*^{F6}/*UAS-AOX*^{F6}; 3/3), *w*^{1118}/*daGAL4* (X/Y; 2/2; *daGAL4*/3), *daGAL4*/*daGAL4* (X/Y; 2/2; *daGAL4*/*daGAL4*), and *w*^{1118} (X/Y; 2/2; 3/3). *Right panel*, The intensity of the AOX bands was determined densitometrically, and normalized (*right panel*) against that of the mitochondrial markers (PDH E1α or ATP5A).

Figure S5. Induction of AOX expression using the *UAS-AOX*^{F6}/*tubGS* system. Protein extracts of crude mitochondrial preparations from whole adult males of the indicated

age and immunoblotting procedure were performed as described in the Materials and Methods. To induce AOX expression, males of the indicated genotype were transferred to new vials containing the inducing drug mifepristone on the day of eclosion, and were cultured for 3 or 10 days before protein preparation. The lines used here were: *UAS-AOX*^{F6}/*w*^{1118} (X/Y; *UAS-AOX*^{F6}/2; 3/3) and *UAS-AOX*^{F6}/*tubGS* (X/Y; *UAS-AOX*^{F6}/2; *tubGS*/3).

Figure S6. Altered testis morphology during adulthood is diagnostic for AOX expression. Mean thickness $\pm$ SD (error bars) of 10-20 dissected (A, C) testes and (B, D) SVs from flies of the ages, genotypes and treatments indicated. Compared with Figure 2, this shows that failure of testis thinning is strictly dependent on AOX expression, whilst abnormal SV morphology can be caused also by the combination of the *tubGS* driver and inducing drug. * indicates statistically significant ($p \leq 0.05$) difference between SVs of induced *tubGS* versus non-induced or non-driver males (B), and between testes (C) and SVs (D) of induced *UAS-AOXwt*$^{8.1}$/*tubGS* versus non-induced or *UAS-AOXmut*/*tubGS* males in either conditions. *UAS-AOXmut*/*tubGS* flies expresses a catalytically deficient form of AOX [27]. Note that the testis morphology of all of the genotypes analysed here is also significantly different from that of AOX-expressing males, as shown in C and D, and in Figure 2 ($p \leq 0.05$). Figure S8B illustrates schematically the thickness measurement protocol used to estimate the amount of immature (in the testes) and mature (in the SVs) sperm cells per fly.

Figure S7. Mature sperm cells accumulate normally in the seminal vesicles (SVs) of males expressing AOX under the control of the *daGAL4* driver. Representative samples of dissected testes and SVs from 10-day old adult males are shown for AOX-expressing

and control lines: *UAS-AOX*^{F6}/*w*^{1118} (X/Y; *UAS-AOX*^{F6}/2; 3/3), *UAS-AOX*^{F6}/*daGAL4* (X/Y; *UAS-AOX*^{F6}/2; *daGAL4*/3), *UAS-GFP*/*w*^{1118} (X/Y; *UAS-StingerGFP*/2; 3/3), and *UAS-GFP*/*daGAL4* (X/Y; *UAS-StingerGFP*/2; *daGAL4*/3). *Red circles* indicate one of the dissected SVs of the representative samples of the indicated genotype. For an explanatory visualization of *D. melanogaster* reproductive organs, see Figure S8A.

Figure S8. Schematic diagrams and light microscopy of *D. melanogaster* male reproductive organs. **A,** *left panel*, illustrating anatomy of the reproductive system, with organs colour-coded to facilitate visualization (adapted from [44]). *Right panel*, light microscopy image of a representative sample from a 6-day-old *w*^{1118} male; colour-matched arrows point to the organs drawn on the *left panel*. The thin and long distal end of the ejaculatory duct is easily ruptured during dissection; therefore, the ejaculatory bulb and external genitalia are rarely present in our images. **B,** light microscopy image of a representative sample from a 3-day-old *w*^{1118} male, with indications of where the thickness of the testis (white arrow) and the seminal vesicle (red arrow) were measured to estimate the amount of immature and mature sperm cells per fly, respectively, as shown in Figure 2C and S6.

Figure S9. AOX is not present in the accessory glands and does not interfere with Sex Peptide production. Immunoflorescence staining and confocal microscopy for AOX (A) and Sex Peptide (B) in dissected accessory glands from a 10-day old male of 3X*tubAOX* (*tubAOX*^{35}/Y; *tubAOX*^{112}/*tubAOX*^{112}; *tubAOX*^{7}/*tubAOX*^{7}) and *w*^{1118} flies. The organ samples were dissected, processed and imaged as described in Materials and Methods, using anti-ATP5A, anti-AOX and anti-Sex Peptide antibodies.

Figure S10. AOX protein is localized in mitochondria of cells from the testis and seminal vesicle (SV) sheaths in *3Xtub-AOX* males. Immunofluorescence staining and confocal microscopy for the indicated proteins and DAPI, from a 10-day old male of genotype *tubAOX*^{35}/Y; *tubAOX*^{112}/*tubAOX*^{112}; *tubAOX*^{7}/*tubAOX*^{7}. AOX protein is localized to the outermost cell layer of these organs, where it overlaps that for the mitochondrial marker ATP5A, but not to germline cells at any stage of differentiation. The light microscopy image (left) is from a control male (*w*^{1118}), used for illustration purpose only. Spermatogenesis takes place inside cysts, starting at the tip of the testes (*upper panels* - note the concentration of nuclei that represent the first stages of this process) and moving towards the proximal end of the organ as cell differentiation proceeds (*middle panels*). At the proximal end of the testis mature spermatozoid cells are released from the cyst and stored in the SV (*lower panels* - note the needle-shaped nuclei that is typical of these cells). See Figure 3 for comparable images from *UAS-AOX*^{F6}/2; *tubGS*/3 males in the presence of mifepristone.

Figure S11. Nuclear and mitochondrial morphologies in germline cells are not altered in 3X*tub-AOX* males. Immunofluorescence staining and confocal microscopy for the indicated proteins and DAPI, from 6-day old *w*^{1118} and 3X*tub-AOX* males, additionally expressing DJ-GFP (**B**, **D**) or not (**A**, **C**): genotypes *w*^{1118}/Y; 2/2; 3/3, *tubAOX*^{35}/Y; *tubAOX*^{112}/ *tubAOX*^{112}; *tubAOX*^{7}/*tubAOX*^{7}, *w*^{1118}/Y; 2/2; 3 DJ-GFP and *tubAOX*^{35}/Y; *tubAOX*^{112}/2; *tubAOX*^{7}/DJ-GFP, respectively). The light microscopy image (*left*) is from a control male (*w*^{1118}), used for illustration purpose only. **A**, Tip of the testis, where first-stage cells are concentrated. Note the agglomeration of nuclei and a more intricate mitochondrial network, as shown by the ATP5A signal. Apart from the expression of AOX, wild-type and *3Xtub-AOX* males are indistinguishable at this stage. **B**, Cysts

containing apolar/mature spermatocytes with dispersed mitochondria throughout the cytoplasm, early spermatids with mitochondria organized as “crescent moon” around the nuclei (only one very early-stage spermatid with mitochondrial aggregation is visible in this field from the *3Xtub-AOX* sample, arrowed), and round spermatids with one huge onion-shaped mitochondrion (the Nebenkern). AOX signal is not observed in this 3X*tubAOX* sample because no ensheathing tissues are shown (see Figures 3 and S10 for AOX signal in this testis region). Note, however, that sperm-tail bundles from later-stage spermatids expressing DJ-GFP, are also visible in this particular field of the *3Xtub-AOX* sample from this region. **C**, Proximal end of the testis, where the needle-shaped nuclei of late spermatid are localized in bundles. Note that this region accumulates the whitish material (Figure 2) and the mature-looking sperm cells (Figure 4) in AOX-expressing males, and starting individualization complexes in males of all genotypes (Figure S14). **D**, Seminal vesicle (SV), where mature spermatozoid cells are stored after spermatogenesis is resumed. Although the SV of AOX-expressing males are smaller (Figure 2 and S6), the mature sperm cells do have normal appearance. Cell differentiation during *Drosophila* spermatogenesis can be visualized in [24].

Figure S12. AOX is expressed in the outermost cell layer of the testis and SV sheaths. **A,** A sample SV from a *UAS-AOX^{F6}*/*tubGS* (X/Y; *UAS-AOX^{F6}*/2; *tubGS*/3) male, under induced conditions, indicating that the inner-cell layer of the sheath manifests ATP5A signal, a mitochondrial marker, but no AOX signal (*grey arrows*), whereas the outermost cell layer, corresponding with the pigment cells, displays both. Testis samples were dissected, processed and imaged as described in the Materials and Methods, using anti-ATP5A and anti-AOX antibodies. The *inset* has a 3X digital zoom. **B,** A sample testis from a *UAS-StingerGFP*/*tubGS* (X/Y; *UAS-StingerGFP*/2; *tubGS*/3)

male, under induced conditions, showing the top view of the proximal end, where the pigment cells tend to concentrate. The *yellow arrows* show examples of bundled needle-shaped nuclei of elongated spermatids (not part of the sheath), whereas the *white arrows* and *ovals* indicate cell nuclei without GFP signal. Because of the size and shape of these nuclei and because of the location of these cells in the inner layer of the sheath, we conclude that these are muscle cells, which are mostly free of AOX.

Figure S13. AOX localizes to the pigment cell layer of the testis and SV sheaths of 3X*tub-AOX* males. **A,** Part of a transverse section of a sample testis (proximal region) stained as indicated for AOX (grey arrows) and for actin fibres, a marker for smooth-muscle cells [43]. Actin is localized more internally in the organ's sheath than the AOX signal. **B,** Part of a transverse section of a sample SV, stained with a mouse antibody for ATP5A and with rabbit antibodies for both AOX and ems, a marker for pigment cells [31, 32]. In addition to mitochondria, brightly staining for AOX and for ATP5A (grey arrows), the ems antibody adds a fainter and more diffuse signal locating to the pigment cells of the outermost layer. Note that the major part of the mitochondrial signal for ATP5A is localized more internally, consistent with a location in the smooth-muscle cell layer. **C**, Partial three-dimensional reconstruction of a sample SV, co-stained with the same antibodies, showing the distribution of signals for ems/AOX and ATP5A in successive layers. Note that the signal for ems and AOX is located more externally than the major ATP5A signal, although mitochondria staining brightly for AOX in the outermost layer are also positive for ATP5A (grey arrows). **D**, Partial three-dimensional reconstruction of a sample testis tip stained for ems and for actin, showing their distribution in successive layers. The ems signal is located externally to that for actin. Testis and SV samples were dissected, processed and imaged as described in the

Materials and Methods, using anti-AOX, anti-ems and anti-ATP5A antibodies, and phalloidin-TRITC.

Figure S14. AOX-expressing males present alterations in sub-testicular structures. **A,** Examples of the structures found throughout the individualization process, the last stage of sperm cell maturation. Testis samples were dissected, processed and imaged as described in the Materials and Methods, using phalloidin-TRITC to detect the actin cones and an antibody to detect activated caspase-3 in the individualization complexes (ICs). Note that the starting ICs are formed when the actin cones are assembled at the base of the late spermatid nuclei (*upper panels*). The apoptotic machinery, including caspase-3, is then recruited to remove the extra cytoplasm and membranes, forming the established ICs (*middle panels*) as they move along the cyst - note the actin cones lined at the equatorial plane of the structure. The final products of this process are individual spermatozoids (not observed here) and waste bags (WBs), which still contain caspase-3 signal and disorganized actin cones (*lower panels*). **B,** Schematic illustration of a control (w^{1118}) male testis, showing the regions where the sub-testicular structures were analyzed. Region 1 and 4 are the distal and proximal end of the testis, respectively; 2 and 3 are the middle region of the organ. **C,** Quantification of the indicated structures per testis region of *UAS-AOX*^{F6}/*tubGS* flies (X/Y; *UAS-AOX*^{F6}/2; *tubGS*/3) of the indicated age in the absence (uninduced) or presence (induced) of 200 μM mifepristone. The data points represent means $\pm$ SD (error bars) of 8-10 individuals analyzed.

Figure S15. Testis malformation in 3X*tub-AOX* males. Representative sample of testis malformation (red arrow) in a 1-day-old adult male of the 3X*tub-AOX* line. Both dissected testes of the same individual are shown.

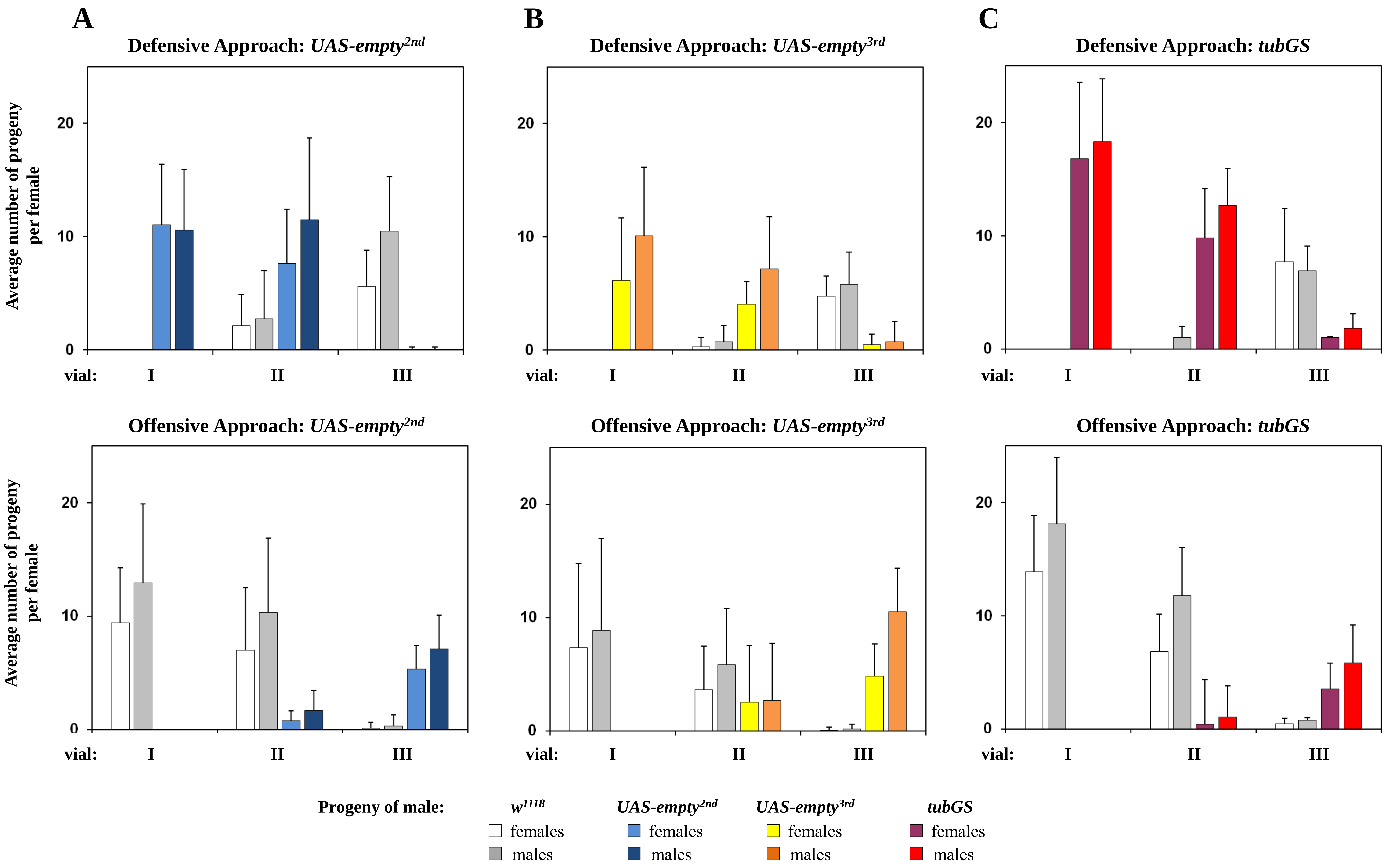

A
Defensive Approach: UAS-empty2nd
B
Defensive Approach: UAS-empty3rd
C
Defensive Approach: tubGS
Offensive Approach: UAS-empty2nd
Offensive Approach: UAS-empty3rd
Offensive Approach: tubGS
Average number of progeny per female
0
10
20
vial:
I
II
III
Progeny of male:
w1118
UAS-empty2nd
UAS-empty3rd
tubGS
females
males

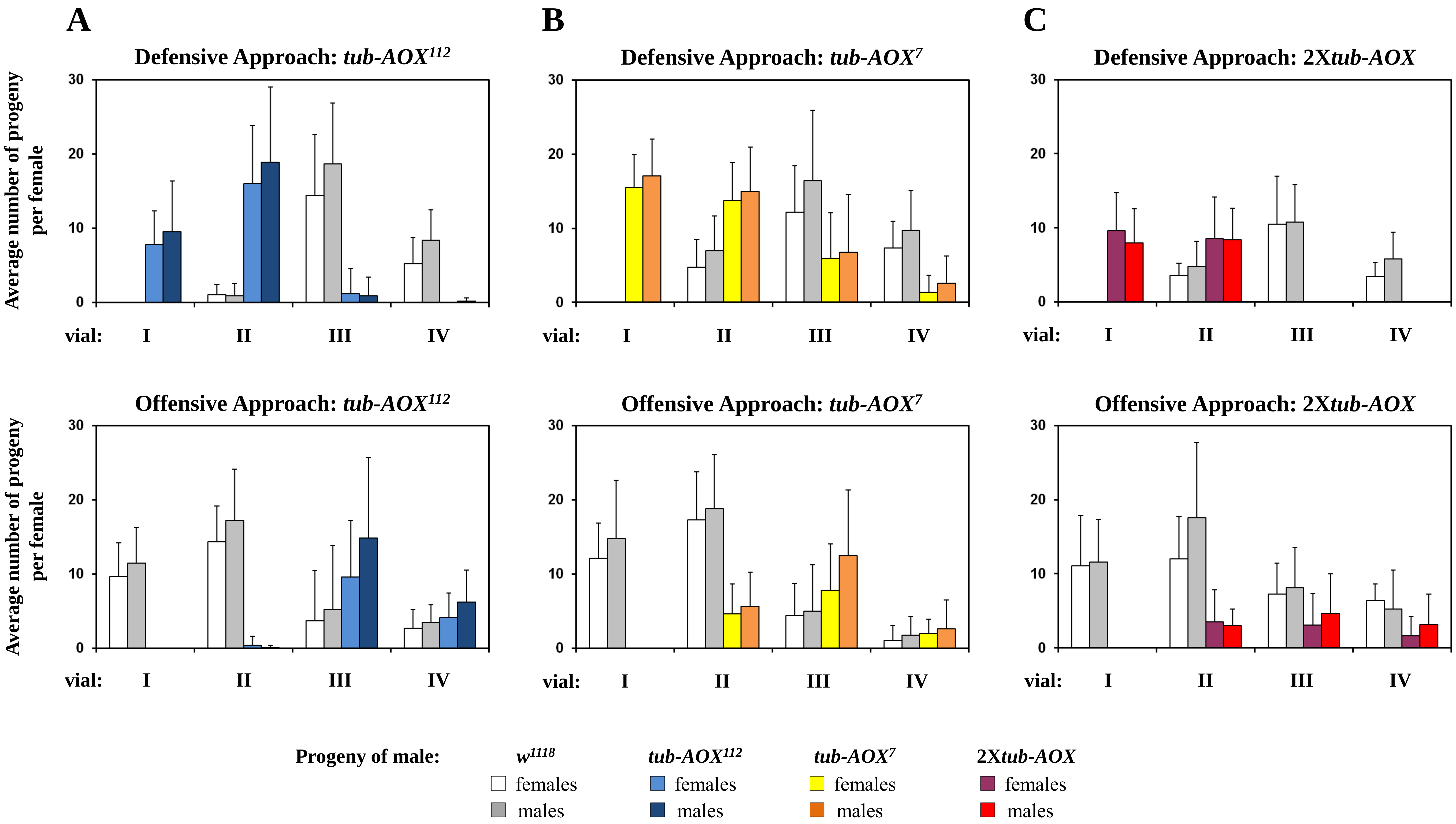
A
Defensive Approach: tub-AOX112
B
Defensive Approach: tub-AOX7
C
Defensive Approach: 2Xtub-AOX
Offensive Approach: tub-AOX112
Offensive Approach: tub-AOX7
Offensive Approach: 2Xtub-AOX
Average number of progeny per female
0
10
20
30
vial:
I
II
III
IV
Progeny of male:
w1118
females
males
tub-AOX112
females
males
tub-AOX7
females
males
2Xtub-AOX
females
males

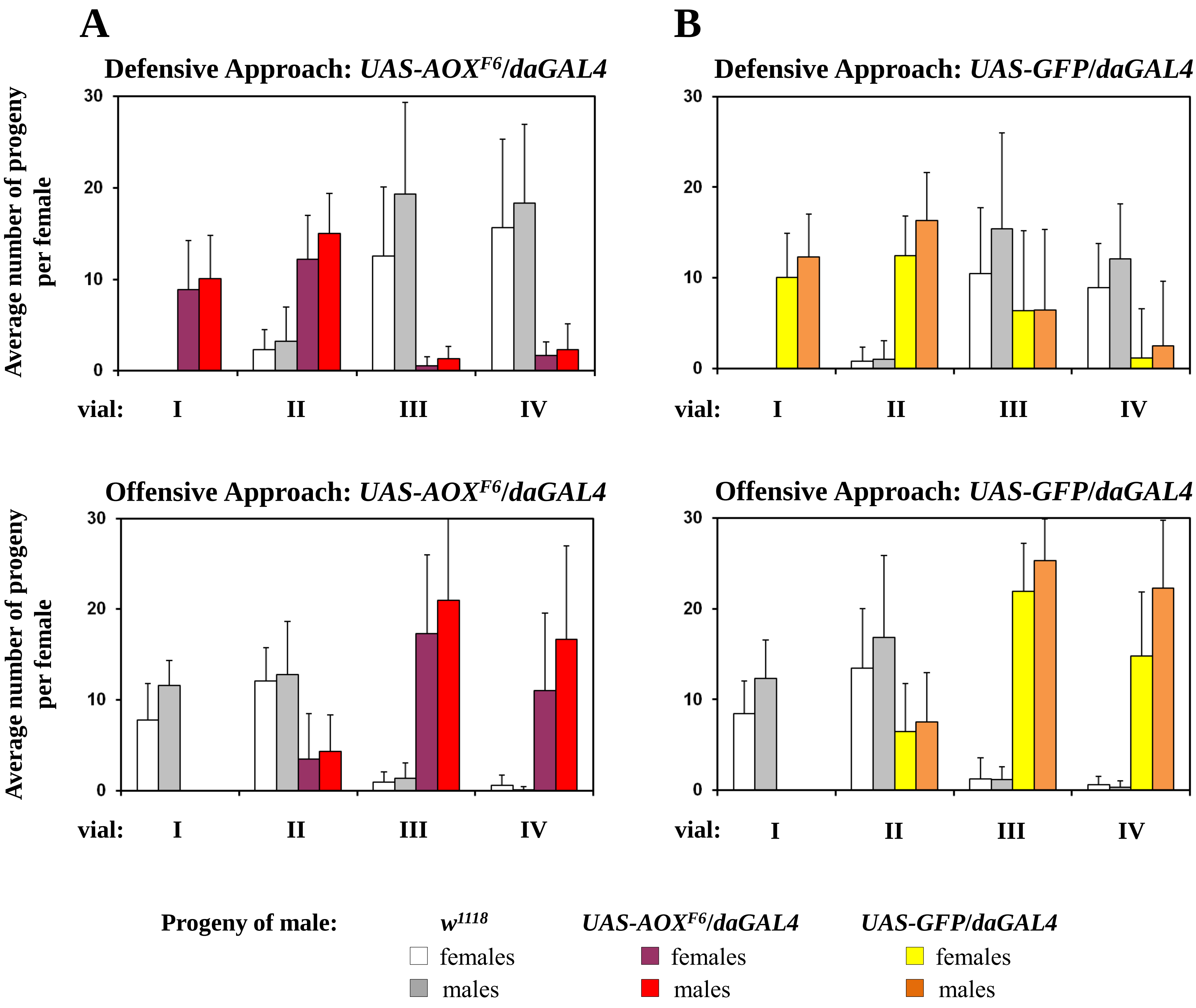

A
B
Defensive Approach: UAS-AOX^F6/daGAL4
Defensive Approach: UAS-GFP/daGAL4
Offensive Approach: UAS-AOX^F6/daGAL4
Offensive Approach: UAS-GFP/daGAL4
Average number of progeny per female
0
10
20
30
vial: I II III IV
Progeny of male:
w1118
females
males
UAS-AOX^F6/daGAL4
females
males
UAS-GFP/daGAL4
females
males

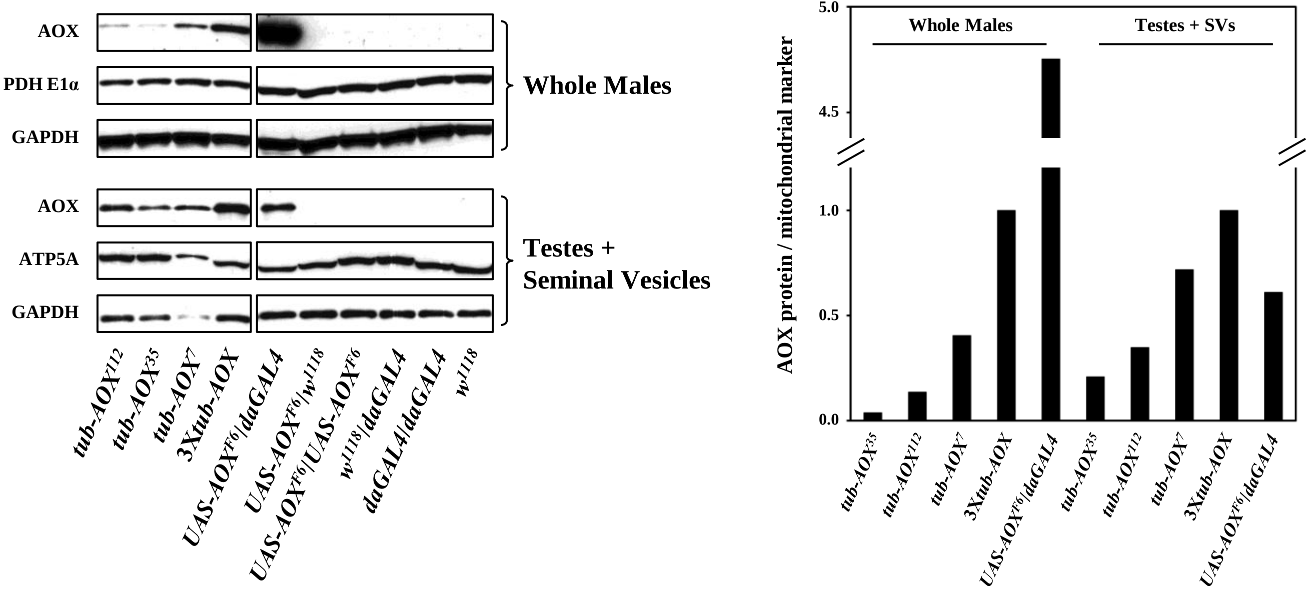
AOX
PDH E1α
GAPDH
Whole Males
AOX
ATP5A
GAPDH
Testes +
Seminal Vesicles
tub-AOX112
tub-AOX35
tub-AOX7
3Xtub-AOX
UAS-AOXF6/daGAL4
UAS-AOXF6/w1118
UAS-AOXF6/UAS-AOXF6
w1118/daGAL4
daGAL4/daGAL4
w1118
AOX protein / mitochondrial marker
5.0
4.5
1.0
0.5
0.0
Whole Males
Testes + SVs
tub-AOX35
tub-AOX112
tub-AOX7
3Xtub-AOX
UAS-AOXF6/daGAL4
tub-AOX35
tub-AOX112
tub-AOX7
3Xtub-AOX
UAS-AOXF6/daGAL4

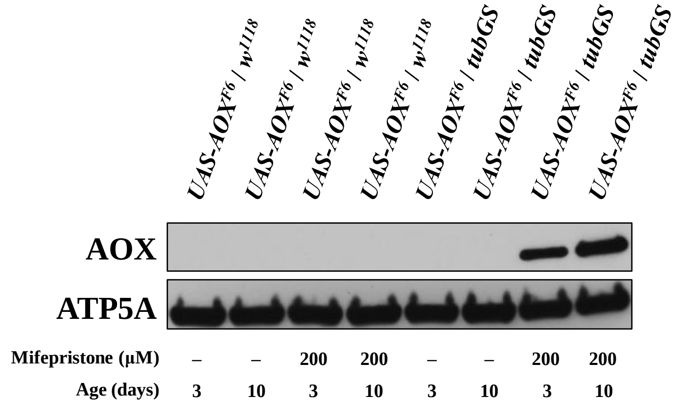
UAS-AOX^F6 / w^1118
UAS-AOX^F6 / w^1118
UAS-AOX^F6 / w^1118
UAS-AOX^F6 / w^1118
UAS-AOX^F6 / tubGS
UAS-AOX^F6 / tubGS
UAS-AOX^F6 / tubGS
UAS-AOX^F6 / tubGS
AOX
ATP5A
Mifepristone (µM) – – 200 200 – – 200 200
Age (days) 3 10 3 10 3 10 3 10

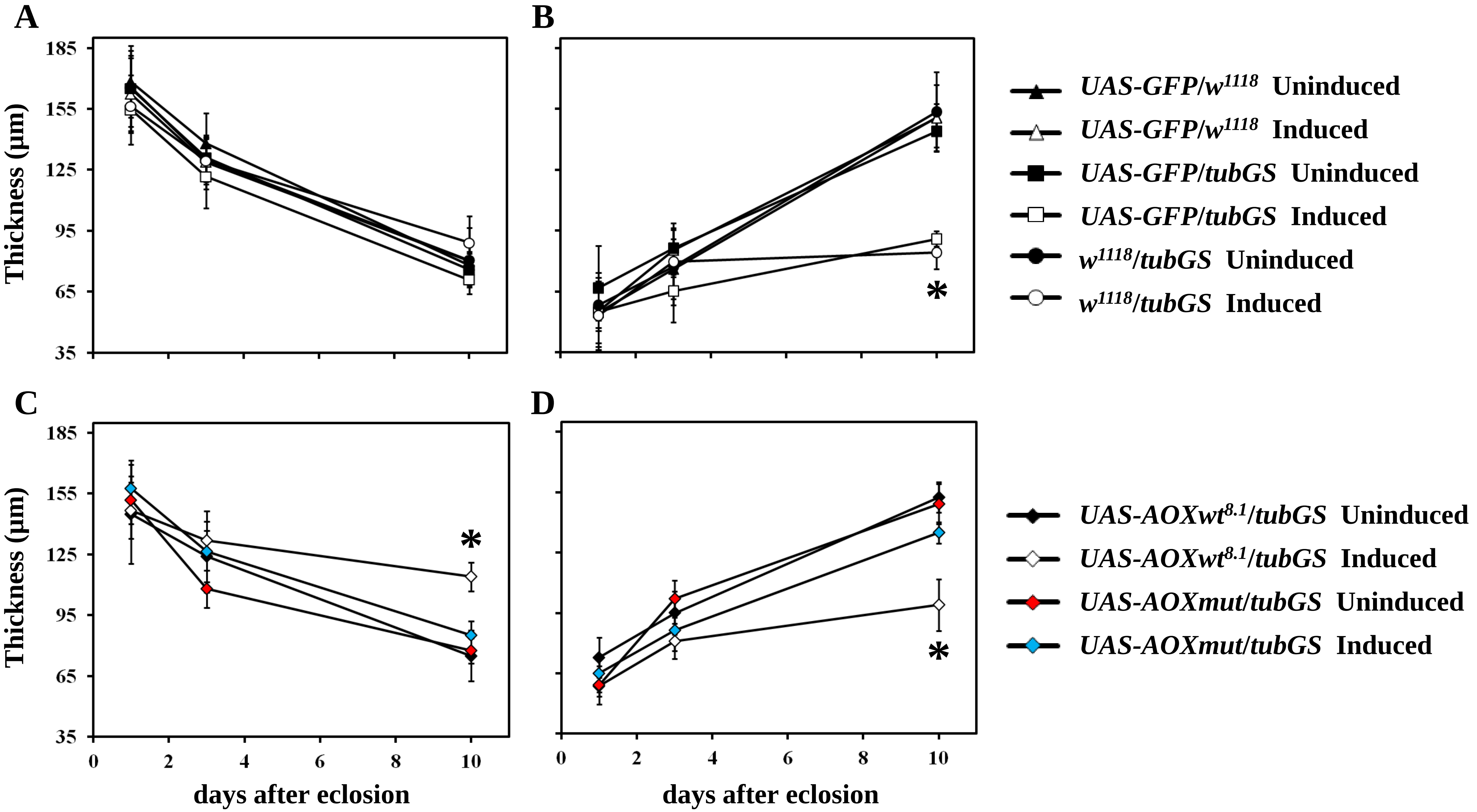

A
B
C
D
Thickness (µm)
185
155
125
95
65
35
0
2
4
6
8
10
days after eclosion
*
UAS-GFP/w1118 Uninduced
UAS-GFP/w1118 Induced
UAS-GFP/tubGS Uninduced
UAS-GFP/tubGS Induced
w1118/tubGS Uninduced
w1118/tubGS Induced
UAS-AOXwt8.1/tubGS Uninduced
UAS-AOXwt8.1/tubGS Induced
UAS-AOXmut/tubGS Uninduced
UAS-AOXmut/tubGS Induced

UAS-AOX^{F6} / w^{1118}

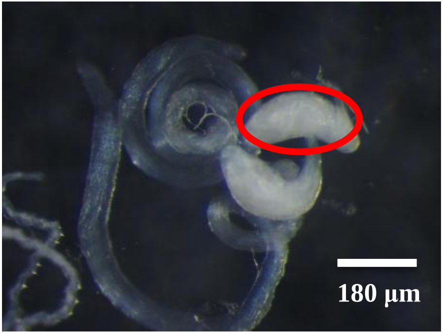

UAS-AOX^{F6} / daGAL4

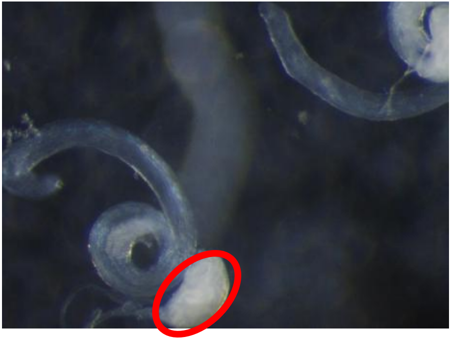

UAS-GFP / w^{1118}

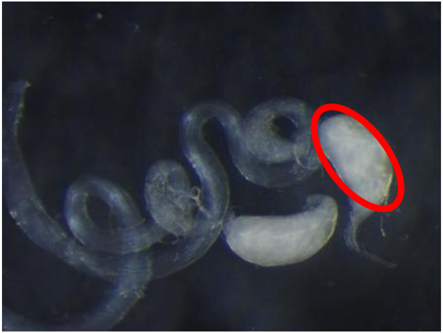

UAS-GFP / daGAL4

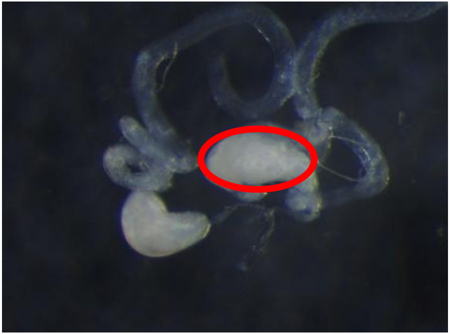

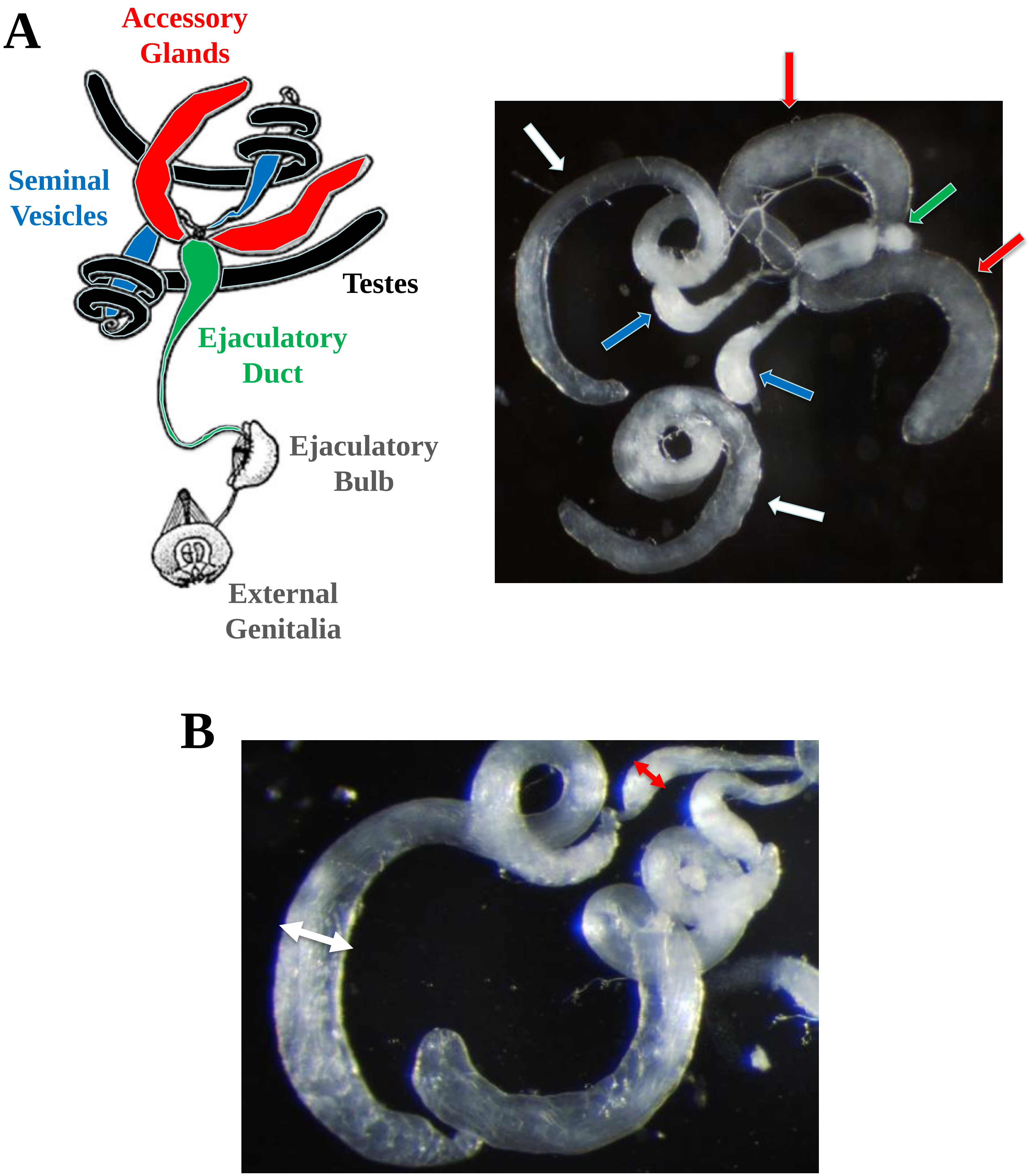
A
Accessory
Glands
Seminal
Vesicles
Testes
Ejaculatory
Duct
Ejaculatory
Bulb
External
Genitalia
B

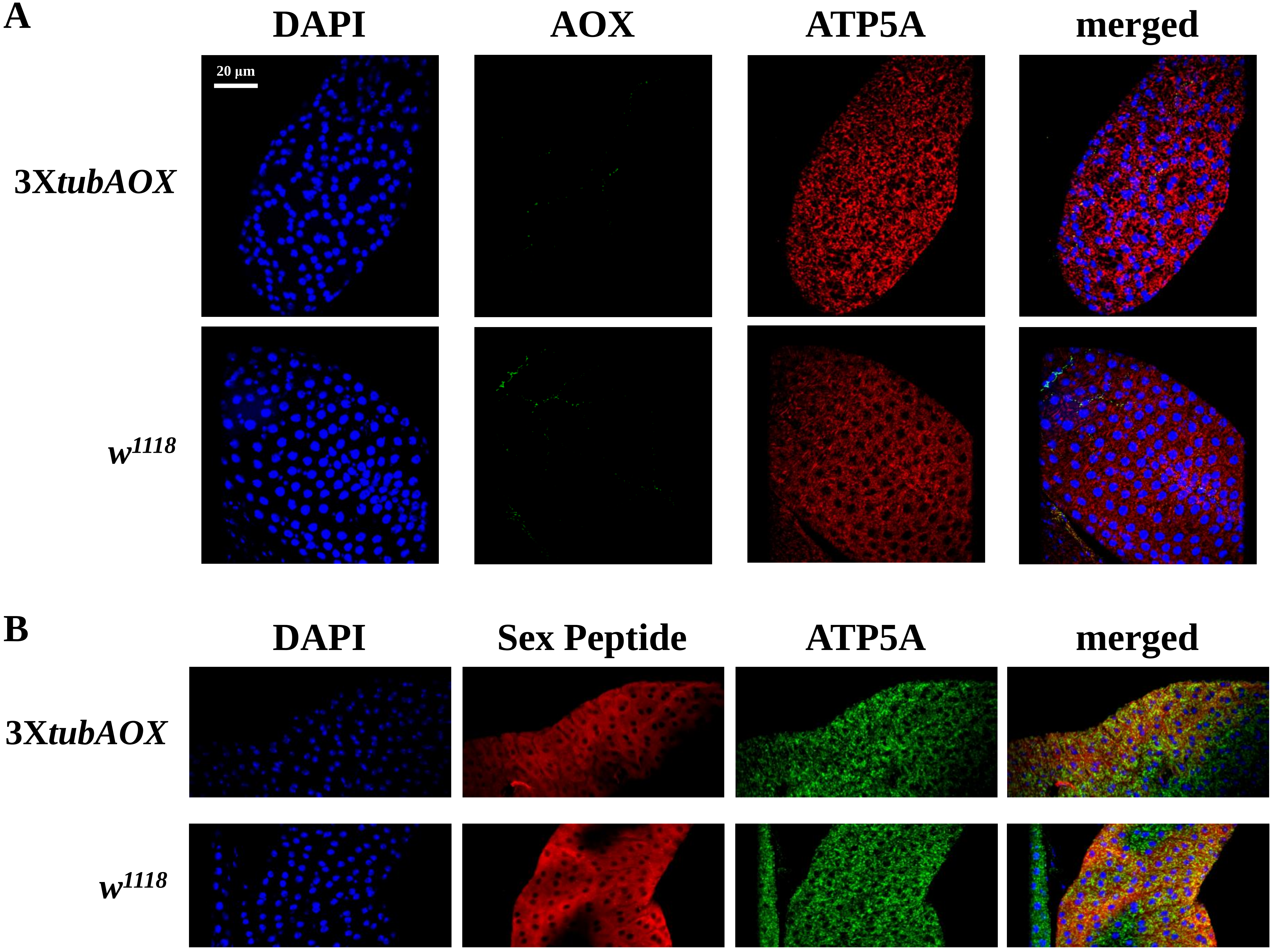
A
DAPI
AOX
ATP5A
merged
20 μm
3XtubAOX
w1118
B
DAPI
Sex Peptide
ATP5A
merged
3XtubAOX
w1118

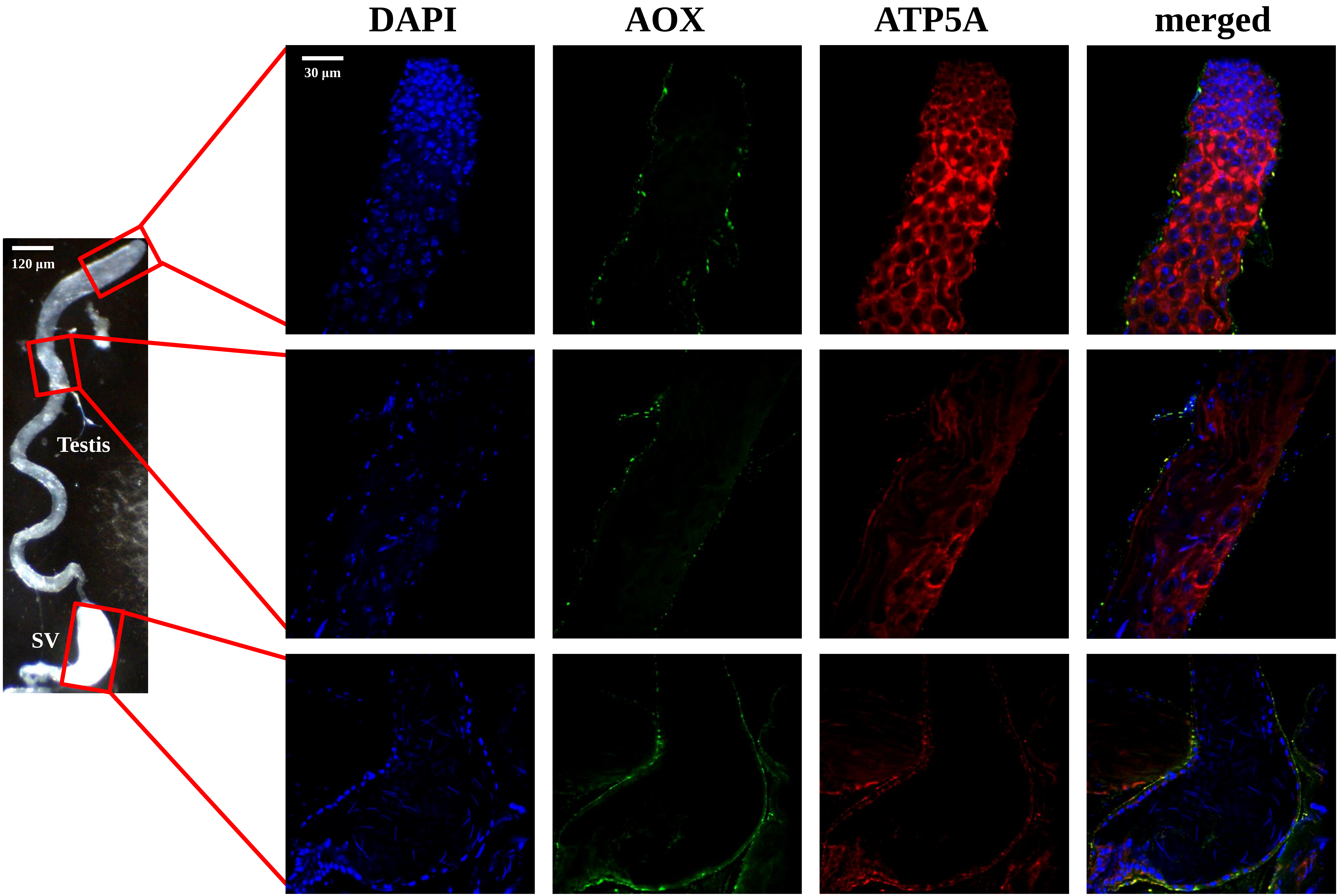
DAPI
AOX
ATP5A
merged
30 µm
120 µm
Testis
SV

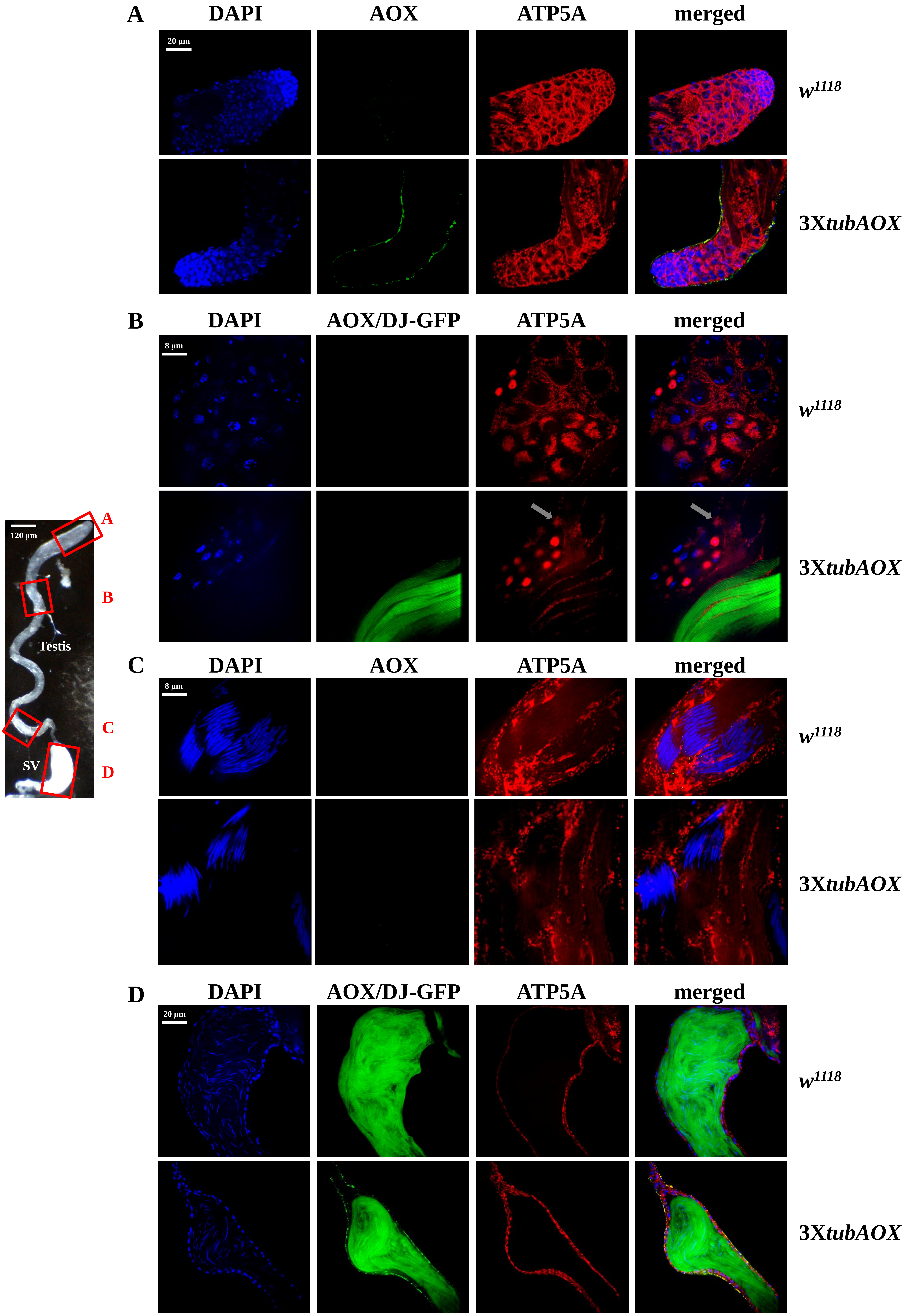
A
DAPI
AOX
ATP5A
merged
20 µm
w1118
3XtubAOX
B
DAPI
AOX/DJ-GFP
ATP5A
merged
8 µm
w1118
3XtubAOX
C
DAPI
AOX
ATP5A
merged
8 µm
w1118
3XtubAOX
D
DAPI
AOX/DJ-GFP
ATP5A
merged
20 µm
w1118
3XtubAOX
120 µm
A
B
Testis
C
SV
D

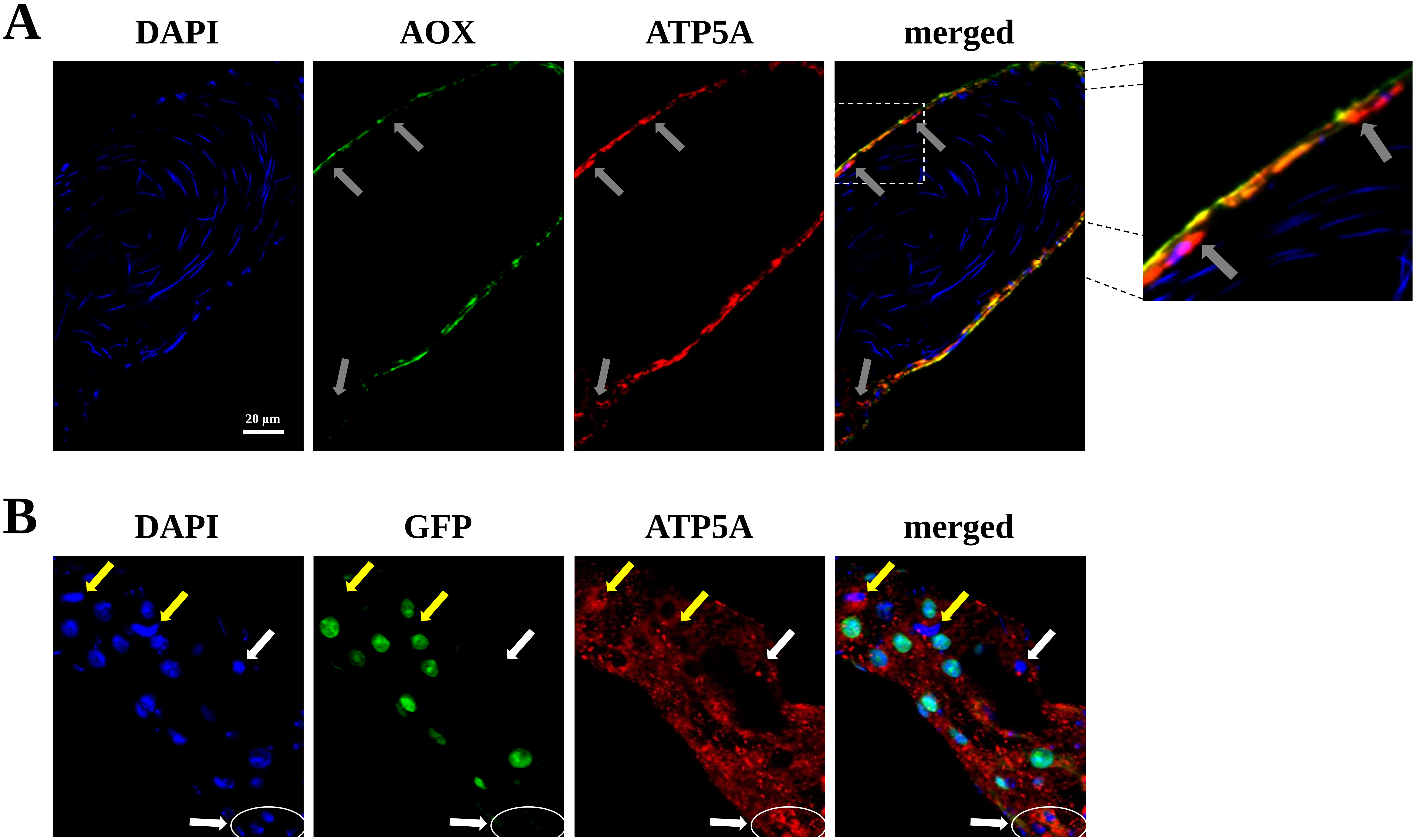
A
DAPI
AOX
ATP5A
merged
20 µm
B
DAPI
GFP
ATP5A
merged

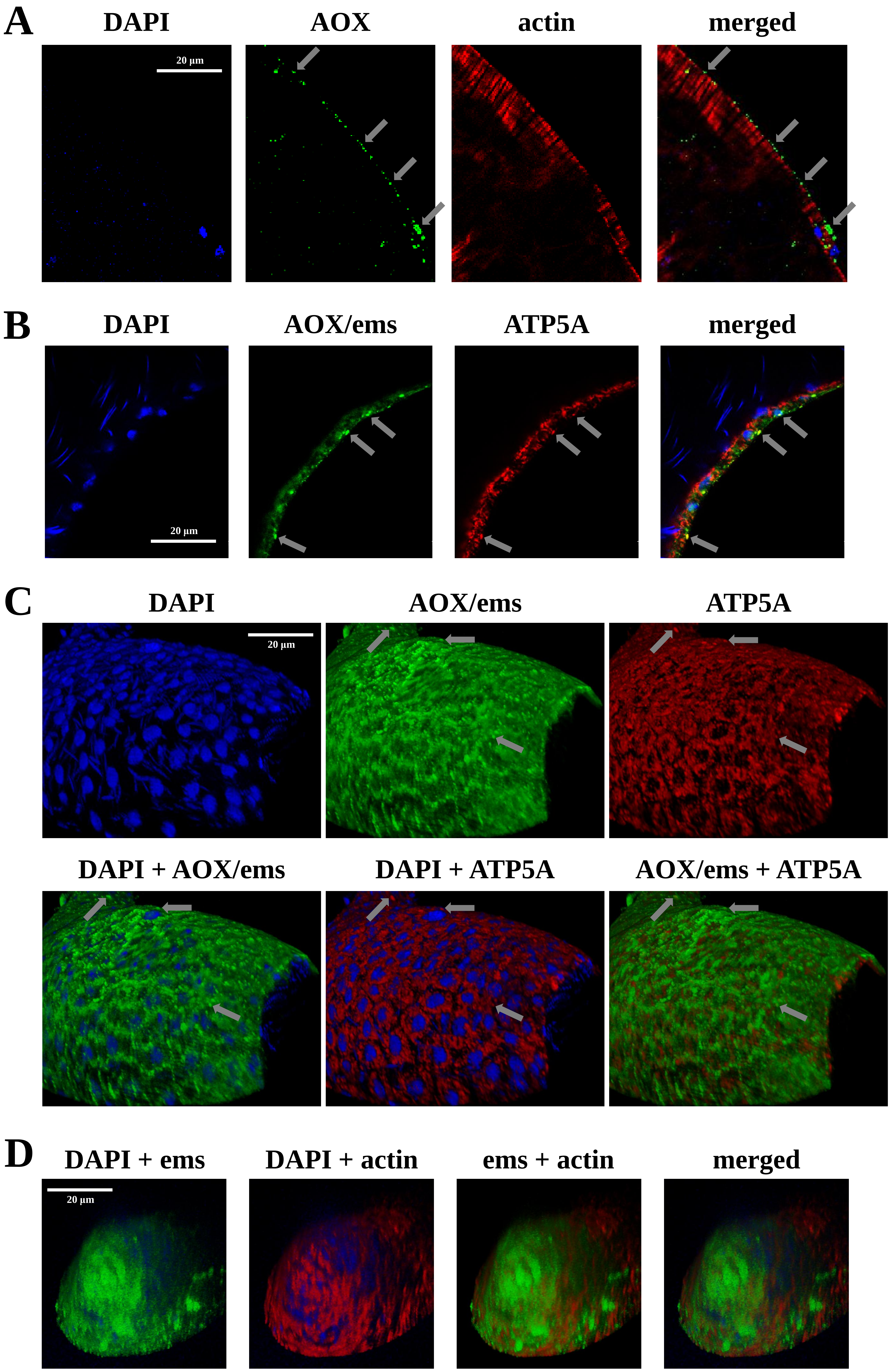
A
DAPI
AOX
actin
merged
20 µm
B
DAPI
AOX/ems
ATP5A
merged
20 µm
C
DAPI
AOX/ems
ATP5A
20 µm
DAPI + AOX/ems
DAPI + ATP5A
AOX/ems + ATP5A
D
DAPI + ems
DAPI + actin
ems + actin
merged
20 µm

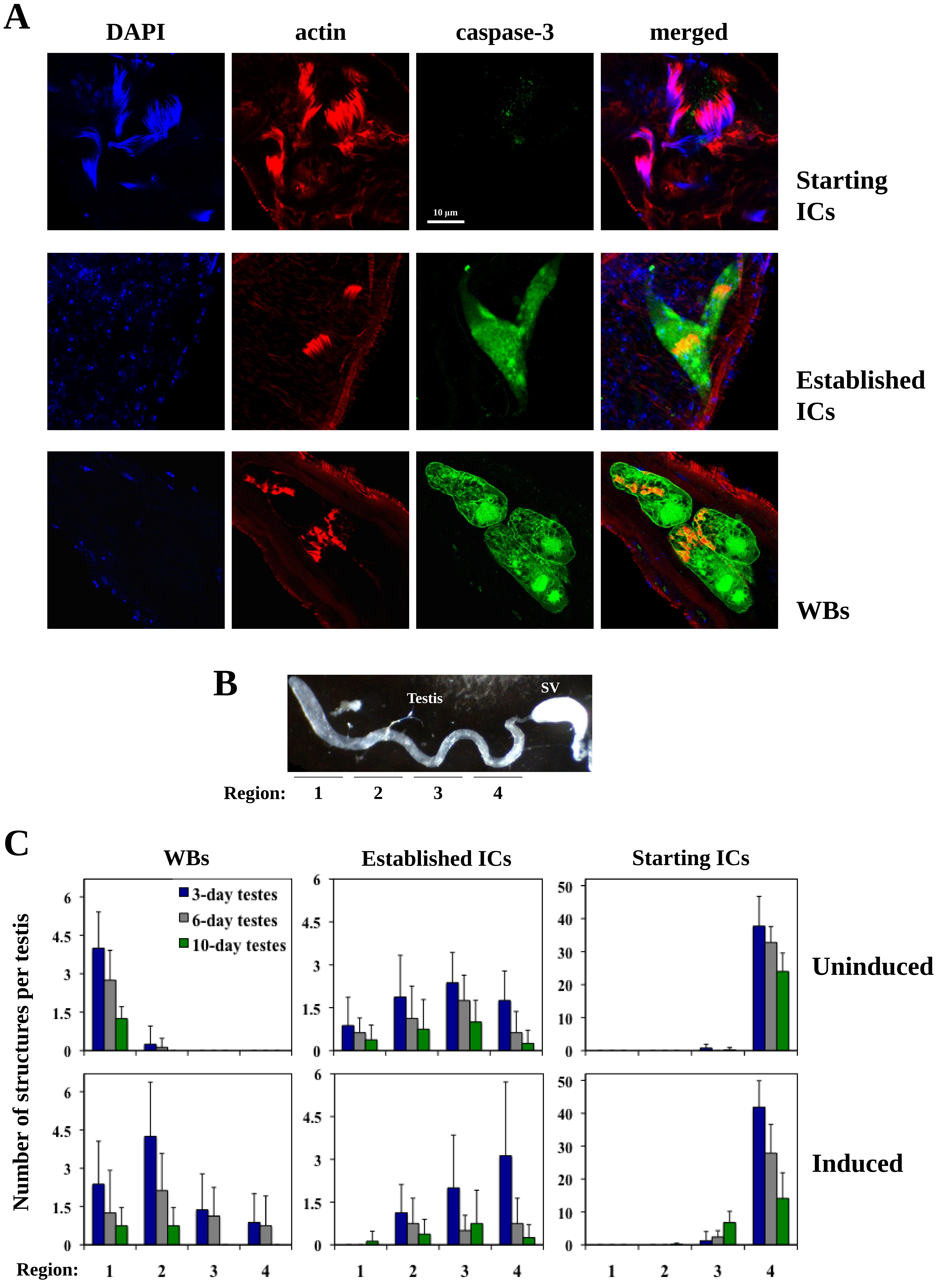
A
DAPI
actin
caspase-3
merged
10 µm
Starting ICs
Established ICs
WBs
B
Testis
SV
Region: 1 2 3 4
C
WBs
Established ICs
Starting ICs
3-day testes
6-day testes
10-day testes
Number of structures per testis
Uninduced
Induced
Region: 1 2 3 4

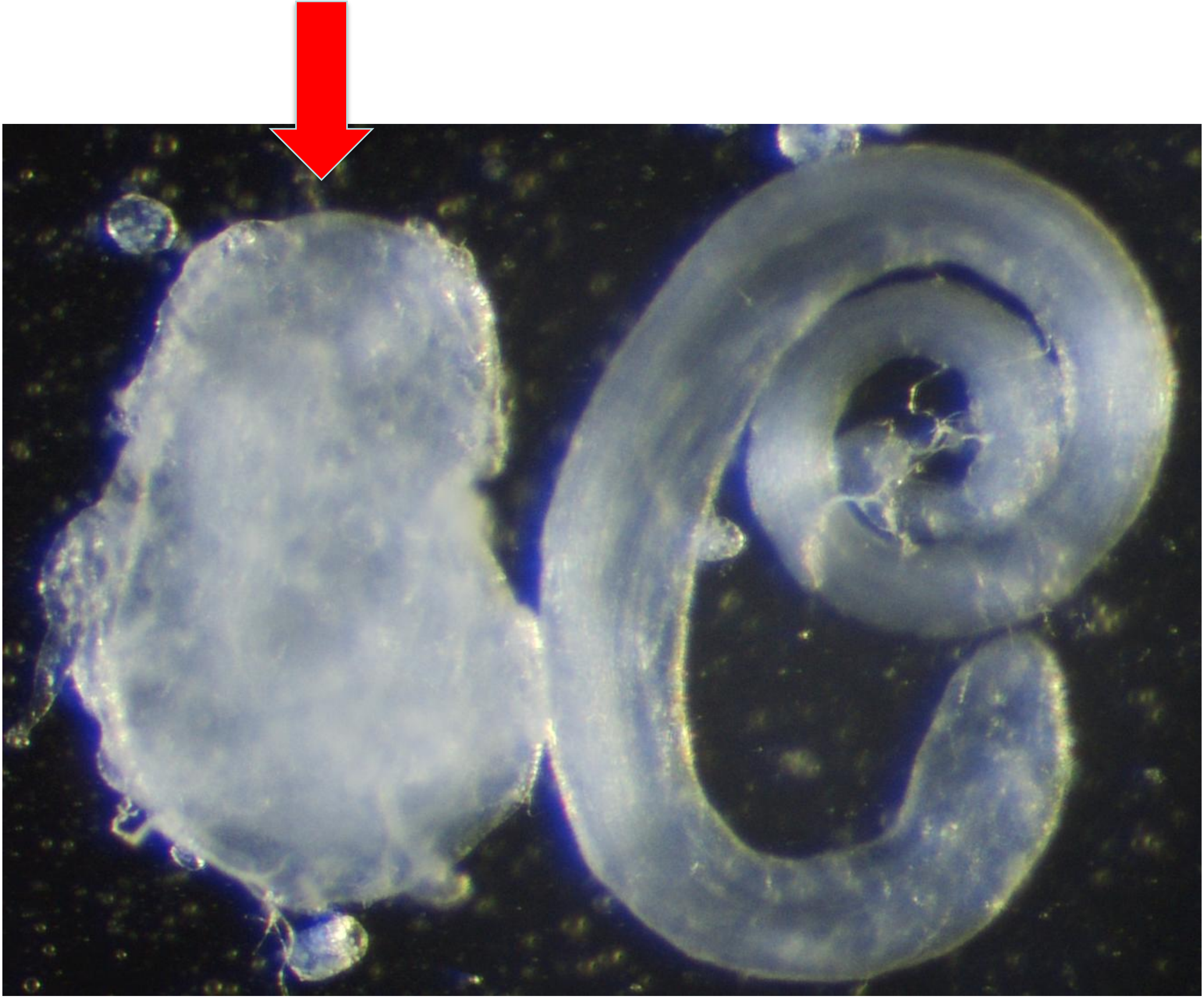

Supplement: Additional file 1: Table S1. — Proportion of progeny sired by the red-eyed male when this is the first male to mate in sperm competition assays against w 1118 males (P1´). Table S2. Proportion of progeny sired by the red-eyed male when this is the second male to mate in sperm competition assays against w 1118 males (P2´). Figure S1. The sperm of eye-color control and tubGS males shows normal competitive properties. Figure S2. The defect in sperm-competition assays is AOX dose-dependent. Figure S3. AOX expressed under the daughterless promoter does not decrease sperm competitiveness. Figure S4. AOX protein levels in testes and seminal vesicles of diverse AOX-expressing lines. Figure S5. Induction of AOX expression using the UAS-AOX F6/tubGS system. Figure S6. Altered testis morphology during adulthood is diagnostic for AOX expression. Figure S7. Mature sperm cells accumulate normally in the seminal vesicles (SVs) of males expressing AOX under the control of the daGAL4 driver. Figure S8. Schematic diagrams and light microscopy of D. melanogaster male reproductive organs [43]. Figure S9. AOX is not present in the accessory glands and does not interfere with Sex Peptide production. Figure S10. AOX protein is localized in mitochondria of cells from the testis and seminal vesicle (SV) sheaths in 3Xtub-AOX males. Figure S11. Nuclear and mitochondrial morphologies in germline cells are not altered in 3Xtub-AOX males. Figure S12. AOX is expressed in the outermost cell layer of the testis and SV sheaths. Figure S13. AOX localizes to the pigment cell layer of the testis and SV sheaths of 3Xtub-AOX males [44]. Figure S14. AOX-expressing males present alterations in sub-testicular structures. Figure S15. Testis malformation in 3Xtub-AOX males. (PDF 3255 kb) [file 12861_2017_151_MOESM1_ESM.pdf]
